# Validating digital polymerase chain reaction for 16S rRNA gene amplification from low biomass environmental samples

**DOI:** 10.1093/ismeco/ycaf115

**Published:** 2025-07-09

**Authors:** Veronika V Koziaeva, Katja Engel, Josh D Neufeld

**Affiliations:** Department of Biology, University of Waterloo, Waterloo, ON N2L 3G1, Canada; Department of Biology, University of Waterloo, Waterloo, ON N2L 3G1, Canada; Department of Biology, University of Waterloo, Waterloo, ON N2L 3G1, Canada

**Keywords:** digital polymerase chain reaction, quantitative real-time PCR, low-biomass samples, PCR contamination, PCR inhibition, DNA quantification

## Abstract

Digital polymerase chain reaction (dPCR) is a DNA quantification technology that offers absolute quantification of DNA templates. In this study, we optimized and validated a chip-based dPCR EvaGreen assay with commonly used 16S rRNA gene primer pairs and compared its performance to quantitative real-time PCR (qPCR). We compared measurements of low amounts of template DNA using a newly designed synthetic DNA standard to assess precision, accuracy, and sensitivity. Optimization approaches were tested to minimize partitions with intermediate fluorescence levels between true positive and true negative partitions (so-called “rain”) for dPCR. Both dPCR and qPCR demonstrated similar quantification performance, with variability in accuracy increasing for samples containing fewer than 30 copies μl^−1^ template concentrations. Both tested 16S rRNA gene primer sets amplified non-target template contaminants within both qPCR and dPCR mixtures, which could not be eliminated by ultraviolet light or DNAse treatment and negatively affected the apparent sensitivity of both PCR assays. Digital PCR was less susceptible to common PCR inhibitors, such as ethanol and humic acids, but was more susceptible to tannic acid inhibition than qPCR. These findings demonstrate the suitability of dPCR for 16S rRNA gene quantification of low biomass environmental samples.

## Introduction

Precise and highly sensitive quantification of microbial DNA is important for assessing microbial dynamics in environmental samples, including those from low-biomass habitats [1]. Extraction of nucleic acids and subsequent quantitative real-time PCR (qPCR) and digital PCR (dPCR) approaches have high analytical sensitivity, specificity, and dynamic range [2], and thus enable measurement of microbial community template abundances, especially in comparison to conventional methods that include counting of colony-forming units, measurements of optical density, or flow cytometry of cells [3]. Although qPCR is widely used for environmental microbiology [1], application of dPCR for such studies has only begun relatively recently following technological advances in microfluidics engineering and emulsion chemistry [4, 5].

Quantitative real-time PCR estimates the concentration of target DNA in a sample by measuring fluorescence in real-time, compared to serial dilutions of a standard template with known concentration [6]. Detecting amplicons during the early exponential phase of PCR enables quantification of gene (or transcript) abundance because these are directly proportional to the initial template concentration [7]. In contrast, dPCR is based on fluorometric end-point PCR. The dPCR mixture is distributed across thousands of individual compartments (from here on referred to as “partitions”) in which the template is randomly distributed. After amplification, the target molecule is measured in each partition based on fluorescence, resulting in positive (target molecule present) or negative (no target molecule present) partitions. Because the occurrence of targets within partitions is random, the resulting data follow a Poisson distribution, enabling calculation of DNA copy numbers without a standard curve [8]. Various dPCR platforms are available, using two main partitioning techniques: droplet-based (e.g. Bio-Rad) and chip-based/microfluidic methods (e.g. Qiagen). Evidence suggests that dPCR results are more affected by variations in DNA extraction methods, as well as differences in PCR mixes used, than by the dPCR platform itself [9].

Preparation of standard curves for qPCR can be time-consuming and errors in calibration curve preparation and differences in amplification efficiencies between reference and environmental templates can introduce bias in the quantification of targets [10–12]. Because dPCR relies on counting of positive and negative reaction partitions, it often yields more reproducible results than qPCR and may lead to more consistent measurements across different laboratories [8, 13–16]. However, both dPCR and qPCR are susceptible to variability introduced by upstream sample handling, including subsampling and pipetting differences [17]. Several studies showed less sensitivity of dPCR to inhibitors, which provides an advantage over qPCR [18–21]. However, dPCR can be affected by inhibitors [22–24]. More systematic studies are needed to assess the effects of environmentally relevant inhibitors for dPCR platforms.

Samples with low biomass are particularly susceptible to contamination because the proportion of contaminating microbial DNA increases with decreasing biomass of the sample [25]. Contaminants can originate from lab surfaces, lab personnel, supplies, or even from ultra-clean certified DNA-free reagents [26, 27]. Because contaminants can be introduced at every stage of a protocol, it is essential to include controls for sampling, DNA extraction, and PCR amplification steps [28, 29]. The accuracy and sensitivity of dPCR or qPCR assays for low biomass samples depend on the presence and source of contaminant DNA template. Although mammalian studies may be impacted by human-associated contamination during production of supplies and reagents, 16S rRNA gene-based studies are impeded strongly by microbial contaminants. In addition to contamination from supplies and lab personnel, the heterologous expression of recombinant enzymes in bacteria, yeast, or plants is a common source for microbial contamination, even for ultra-pure enzymes [30–37].

Although dPCR has gained popularity in clinical research, most applications to date have relied on droplet-based platforms, TaqMan probe chemistry, and target-specific primer sets. In contrast, universal 16S rRNA primers are widely used in microbial ecology but have not been validated systematically for use with chip-based dPCR systems for routine quantification of low-biomass, mixed-community samples. In this study, the evaluation of performance and optimization of a chip-based dPCR EvaGreen assay with universal 16S rRNA primers were performed across a range of template concentrations. For that, a synthetic DNA standard was used to compare the performance of dPCR and qPCR using primers targeting the 16S rRNA gene, specifically 341F-518R and 515F-Y–806R, which are widely used for quantification of total bacterial or prokaryotic DNA, respectively. In addition, this synthetic template contained P5-P7 primer binding sites, which enabled the assessment of quantification accuracy because these primers have no or very low matches to genomic sequences and thus are not sensitive to reagent contamination. Several amplification conditions were tested to assess impacts on PCR efficiency and quality. Furthermore, inhibition of dPCR and qPCR was tested for PowerMax Soil DNA kit extracts, ethanol, and humic and tannic acids.

## Materials and methods

### Synthetic DNA standard

A fragment containing the full length *Thermus thermophilus* 16S rRNA gene sequence fused downstream to a partial beta-glucuronidase enzyme (*uidA)* gene from *Escherichia coli* was synthesized and cloned into pUC57-Kan vector (Suppl. Fig. 1) by Bio Basic (Ontario, Canada). The 165-nucleotide *uidA* gene fragment was flanked by primers P5 and P7. The primer pair M13F and M13R (Messing, 1983) flanks the *uidA*-16S rRNA gene fragment and was used to generate synthetic standard template for dPCR validation. All primer sequences used in this study (Table 1) were purchased from Sigma-Aldrich (Burlington, MA, USA).

**Table 1:** Primers used for dPCR validation.

Target	Primer	Sequence (5′-3′)	T_m_	Nucleotides	Reference
*uidA*	P5	AATGATACGGCGACCACCGAGAT	60	165	[38]
	P7	CAAGCAGAAGACGGCATACGA			
16S rRNA	515F-Y	GTGYCAGCMGCCGCGGTAA	50	292	[39]
	806R^*^	GGACTACHVGGGTWTCTAAT			[40]
16S rRNA	341F	CCTACGGGAGGCAGCAG	55	194	[41]
	518R	ATTACCGCGGCTGCTGG			
Synthetic standard	M13F	GTAAAACGACGGCCAG	50	1720	[42]
	M13R	CAGGAAACAGCTATGAC			

^*^The revised 806R primer sequence from Apprill et al. 2015 (GGACTACNVGGGTWTCTAAT) may be preferrable when studying marine and lake environments.

### Synthetic DNA standard template preparation

Using the cloned synthetic fragment as template (10 ng), the M13F and M13R primer pair was used to amplify the standard in a 50 μl PCR with 1X ThermoPol Reaction Buffer (New England Biolabs, Ipswich, MA, USA), 0.2 mM dNTP (New England Biolabs), 0.2 μM each primer, 0.6 mM BSA (Sigma-Aldrich), 0.25 U *Taq* polymerase (New England Biolabs), and HyPure nuclease-free water (Cytiva, Marlborough, MA, USA). The amplification protocol consisted of initial denaturation at 95°C for 5 min followed by 35 cycles of denaturation at 95°C for 30 s, annealing at 50°C for 30 s, elongation at 68°C for 1 min, followed by a final elongation step at 68°C for 7 min. The resulting PCR product was purified using the Wizard SV Gel and PCR Clean-Up kit (Promega, Madison, WI, USA). The concentration of purified synthetic standard was measured using Qubit dsDNA High Sensitivity Assay Kit (Invitrogen, Waltham, MA, USA). Further, the synthetic standard was diluted to 2 ng μl^−1^ (~1 × 10^9^ copies μl^−1^) in TE buffer with Tween 20 and stored at −20°C in single-use aliquots to avoid freeze–thaw degradation. Thawed synthetic standard was quantified using the Qubit dsDNA High Sensitivity Assay Kit (Invitrogen) and serial decimal dilutions were prepared in RNase-free water derived from the dPCR kit (Qiagen, Germany).

### dPCR and qPCR protocols

All PCR setups were performed in a PCR Workstation (AirClean Systems, Creedmoor, NC, USA) with ISO 5-HEPA-filtered air and surfaces treated with UV light for 15 min before each use. Tubes and PCR plates were UV treated (365 nm) for 20 min on a transilluminator (ProteinSimple, San Jose, CA, USA). The qPCR assay was performed on a CFX Opus System (Bio-Rad, Hercules, CA, USA) using the SsoAdvanced Universal SYBR Green Supermix (1725271, Bio-Rad). Two μl of synthetic standard tested in 15 μl reaction mixtures consisted of 1X SsoAdvanced Universal SYBR Green Supermix (Bio-Rad) with a final concentration of 0.3 μM for each primer. In addition, a final concentration of 0.5 mg ml^−1^ BSA was added to the qPCR mixture. Triplicate reactions were used to generate a standard curve using a decimal dilution of the synthetic standard from 1 × 10^6^ to 1 × 10^1^ copies μl^−1^. Thermocycling conditions for P5-P7 primer pair included initial denaturation at 98°C for 3 min, and 40 cycles of denaturation at 98°C for 15 s, and annealing at 60°C for 30 s. Thermocycling conditions for both 16S rRNA gene primer pairs included a three-step protocol: 40 cycles of denaturation at 98°C for 15 s, annealing at 50°C or 55°C (Table 1) for 15 s, and elongation at 72°C for 30 s.

The dPCR assay was conducted on the QIAcuity Digital PCR System (Qiagen) using the QIAcuity EvaGreen (EG) PCR Kit (250111, Qiagen). The dPCR assay used the same primers as qPCR. Reaction mixtures (15 μl) contained QIAcuity EvaGreen Mastermix (Qiagen), 400 nM each primer, and 2 μl of the synthetic standard. No template digestion or shearing was conducted. For all analyses, 8.5k dPCR nanoplates (Qiagen) were used with 12 μl reaction mixtures. A dPCR nanoplate preparation was done following the manufacturer’s protocol. Thermocycling conditions were the same as for qPCR, except 95°C was used for denaturation and the initial denaturation step was for 2 min. The QIAcuity Software Suite (version 2.5) was used to analyse dPCR results and calculate copy numbers. A common threshold was manually set to separate positive and negative partitions. Copy number for dPCR and qPCR was calculated per μl of synthetic standard added.

### Testing PCR parameters and precision

To access the accuracy and precision of dPCR and qPCR, serial dilutions of the synthetic standard were used in a range of 1 × 10^3^ to 1 copies μl^−1^. The synthetic standard (2 μl) was added to each reaction in triplicate. Triplicate no-template controls (NTCs) were included in each nanoplate or 96-well plate and for each primer pair. Average NTC copy numbers were subtracted from samples.

The effect of PCR cycle number on dPCR quantification was evaluated to determine a better resolution of positive and negative partitions for 16S rRNA gene amplifications and to reduce intermediate (“rain”) partitions (Suppl. Fig. 2). A nanoplate was quantified after 40 and 50 PCR cycles. Furthermore, the effect of primer concentration in a range from 100 to 500 nM was tested in duplicate with 1 × 10^4^ copies μl^−1^ of synthetic standard and corresponding NTCs.

To assess precision for low copy number samples, 2, 4, and 8 μl of the synthetic standard were tested by dPCR in triplicate. The synthetic standard concentrations used were 30, 10, and 3 copies μl^−1^. The nanoplate was quantified after 40 and 50 PCR cycles.

### Statistical analyses

Weighted linear regression (WLR) was used to assess dPCR and qPCR measurements [43]. Reciprocals of variance were used as weights, and calculations for 95% estimation confidence intervals (CIs) were performed. WLR lines were fitted directly to the raw data values and plotted on logarithmic axes. The use of logarithmic scaling highlights patterns across multiple orders of magnitude without applying a log transformation to the data. A 95% CI was used to compare dPCR and qPCR measurements [44]. The relative standard deviation (RSD, %), equivalent to the coefficient of variation, was used to assess the variability among replicate measurements. The “modified signed-likelihood ratio test for equality of CVs” was used to test for significant differences using the R package cvequality (version 0.2.0) [45, 46]. For predicting variation in target measurements, a weighted linear regression was performed using a model of RSD versus log10 copies μl^−1^. Significant differences for template volume and decontamination experiments were evaluated by pairwise Wilcoxon test with Benjamini-Hochberg correction [47] after Shapiro–Wilk normality test. All statistical tests were performed using base packages in R (http://www.r-project.org/).

### DNA isolation

Blank extractions (i.e., no sample added) were performed with the DNeasy PowerMax Soil DNA Kit (Qiagen) to obtain “kit control” extracts. Extractions were done using the manufacturer’s protocol with several modifications [48]. PowerBead tubes were incubated at 70°C for 10 min followed by beadbeating using an MM 400 Mixer Mill (Retsch, Germany) at 30 Hz for 10 min. The final elution volume was 2 ml. The kit control extractions were performed independently by three researchers who followed the same extraction protocol multiple times on different days and with different lot numbers of DNA isolation kits. Researcher 1 performed three extractions, researchers 2 and 3 each did two extractions. Kit control extracts were stored at −20°C until further analysis.

### Inhibition

The susceptibility of dPCR and qPCR to inhibition was investigated using humic and tannic acids, ethanol, and blank DNA extract (kit control). Two μl of 1 × 10^4^ copies μl^−1^ standard was added to reaction mixtures. The P5-P7 primer pair was used for inhibition tests. Humic acid sodium salt (Sigma-Aldrich, #H16752) and tannic acid (Sigma-Aldrich, #403040) were dissolved in PCR water to prepare 4.8 mg ml^−1^ stock solutions. Stock solutions were diluted and spiked into 15 μl of dPCR and qPCR to achieve final concentrations of 0, 1.5, 3, 6, 12, 25, 50, 100, 200, 400, and 800 ng μl^−1^ in each PCR mixture. Ethanol (Greenfield Global Ink, Canada, #P210EAAN) was diluted and spiked into PCR mixtures to achieve final concentrations of 0,% 1%, 2%, 3%, 4%, 5%, 6%, and 8%. Kit controls were added to 15 μl dPCR mixtures in volumes of 0, 2, 4, 5, 6, 7 μl, which corresponded to 0%, 13%, 27%, 33%, 40%, and 47% spike concentrations. For qPCR, 0%, 13%, 27%, and 33% of kit control spikes were added to each reaction. A half-maximal inhibitory concentration (IC_50_) was determined using the drc package (version 3.0–1) for dose–response modelling [49].

### Decontamination

To quantify and remove potential contamination from PCR water, different types of PCR water and length of ultraviolet (UV) treatment were tested. Tubes (2 ml) of dPCR kit water (Qiagen) from the same lot number were thawed and then exposed to UV (365 nm) for 20 and 40 min on a transilluminator (ProteinSimple). HyPure Molecular Biology Grade Water (Cytiva) was aliquoted from a newly opened bottle into 1.5 ml SafeSeal low-DNA-binding plastic tubes (Sarstedt, Germany, #72.706.700) and then exposed to UV for the same amount of time as kit water. Both dPCR and qPCR were performed using 16S rRNA gene primers. A nanoplate was quantified after 40 and 50 PCR cycles.

DNAse treatment was performed using two units of heat-labile dsDNAse (ArcticZymes, Norway, 70 800–201), which were added to the dPCR mix containing primers, template, and an additional 0.5 μl of 100 mM MgSO_4_. The reaction was incubated at 37°C for 10 min. Subsequently, dithiothreitol (DTT) was added to each sample to a final concentration of 0.001 M, followed by incubation at 70°C for 20 minutes. A 12 μl decontaminated mix was transferred to the dPCR plate.

### Mock community and environmental samples

ZymoBIOMICS Microbial Community DNA Standard (D6305, Zymo Research, USA), groundwater, and bentonite DNA extracts were used for dPCR validation on a defined community and environmental samples. The Microbial Community DNA Standard (“mock community”) consisted of eight bacterial species, each represented with 12% DNA abundance (*Pseudomonas aeruginosa*, *E. coli*, *Salmonella enterica*, *Lactobacillus fermentum*, *Enterococcus faecalis*, *Staphylococcus aureus*, *Listeria monocytogenes*, *Bacillus subtilis*) and two fungal species, each with 2% DNA abundance (*Saccharomyces cerevisiae*, *Cryptococcus neoformans*). The mock community DNA was diluted with RNase-free water derived from the dPCR kit to 8968 and 89.68 copies μl^−1^. DNA from dry (bentonite 1) or hydrated (bentonite 2) Wyoming MX-80 bentonite was extracted using the DNeasy PowerMax Soil DNA Kit (Qiagen) as described above. DNA concentrations of bentonite extracts were below the detection limit of the Qubit dsDNA High Sensitivity Assay Kit (Invitrogen, Waltham, MA, USA). The DNeasy PowerSoil Pro Kit was used to extract DNA from groundwater samples following the manufacturer’s instruction using a bead beater (FastPrep-24 Instrument MP Biomedicals, USA) at 5.5 m/s for 45 s instead of vortex. DNA concentrations were 0.042 (groundwater 1) and 0.084 (groundwater 2) ng μl^−1^ based on the Qubit fluorometer. The groundwater 16S rRNA gene copy numbers were estimated using an average bacterial genome size of 3.65 Mb [50] an average 16S rRNA gene copy number of 3.6 [51] and an average molecular weight of 650 g per mole of double-stranded DNA. The qPCR and dPCR assays were performed in triplicate using the same 16S rRNA gene primers, and cycling conditions as described above and 2 μl template. Digital PCR was performed using 400 nM each primer, and for 40 and 50 cycles. For qPCR and dPCR, the NTC was subtracted from sample values.

## Results

### PCR parameters, accuracy, and precision

**Figure 1:**
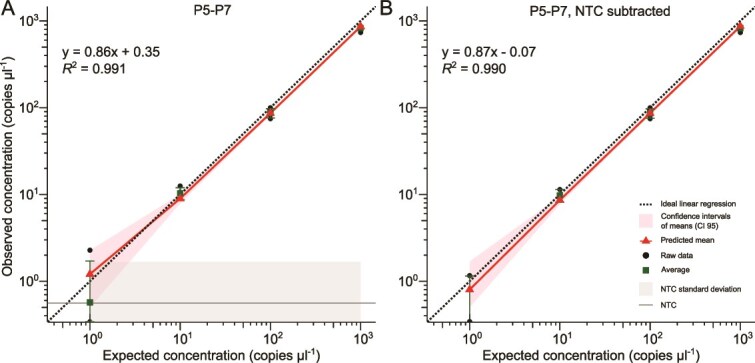
Digital PCR performance using P5-P7 primer pair. Weighted linear regression model illustrates relationship between observed and expected copy number concentration per μl of synthetic standard. The diagonal solid line is the predicted mean concentration, the shadow around the solid line is 95% confidence interval of the means. Dots represent raw data, squares indicate the average concentration with standard deviation. The theoretical optimal quantification is indicated with the dashed line representing identical observed and measured concentrations. Horizontal line indicates average of NTCs. Shadow around the NTC line represents standard deviation.

The performance of dPCR was tested with primers P5-P7 (Table 1) and the synthetic standard as template to determine the ability of dPCR to quantify low copy numbers when the amplification reaction is unaffected by nucleic acid contamination. Based on a WLR model, the quantification values obtained from dPCR were very similar to gene copy numbers derived from Qubit measurements (Fig. 1A). However, at 1 copy μl^−1^, which was the lowest concentration tested, the RSD of replicates was 200%, which was significantly higher than at other concentrations (*P* = 1.88 × 10^−6^) (Suppl. Table 1). At this concentration, the 95% CIs overlapped with those of NTCs, whereas RSD values at higher concentrations were below 15% (Fig. 1A). Average copy number measured for NTCs was 0.6 ± 1.1 copies μl^−1^ (Fig. 1A). We observed good separation of positive and negative partitions with primer P5-P7 amplifications from synthetic standard template, with very few intermediate partitions (“rain”; Suppl. Fig. 3 and Suppl. Fig. 4C). The WLR model, after subtraction of NTC abundances, showed more accurate values and narrower CI ranges, and showed the same pattern in variability of replicates (*P* = 5.31 × 10^−9^) compared to the model based on values with unsubtracted NTCs (Fig. 1B, Suppl. Table 1).

**Figure 2:**
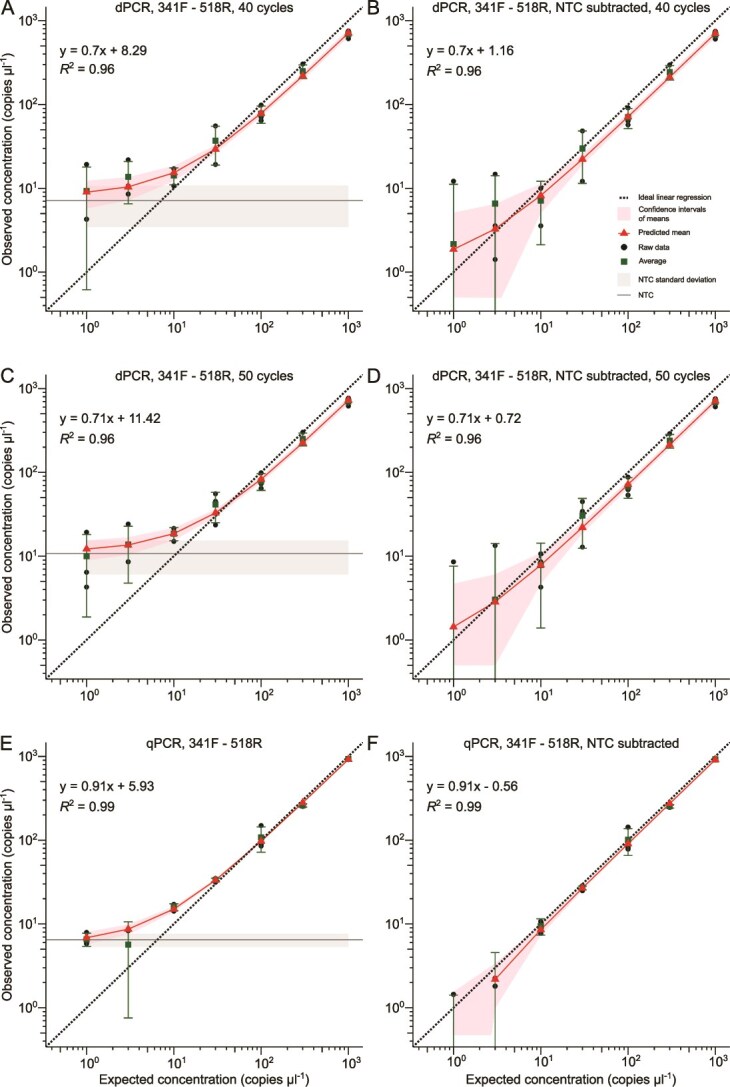
Digital PCR and qPCR performance for 341F-518R primer pair. Weighted linear regression models illustrate relationship between observed and expected copy number concentration per μl of synthetic standard for dPCR after 40 PCR cycles (A), dPCR after 40 cycles with average NTCs subtracted (B), dPCR after 50 PCR cycles (C), dPCR after 50 cycles with subtracted average NTCs (D), qPCR after 40 PCR cycles (E), and qPCR after 40 PCR cycles with subtracted average NTCs (F). The diagonal solid line is the predicted mean concentration, the shadow around the solid line is 95% confidence interval of the means. Dots represent raw data, squares indicate the average concentration with standard deviation. The theoretical optimal quantification is indicated with the dashed line representing identical observed and measured concentrations. Horizontal lines represent average of NTCs. Shadow around NTC lines represents standard deviation.

**Figure 3:**
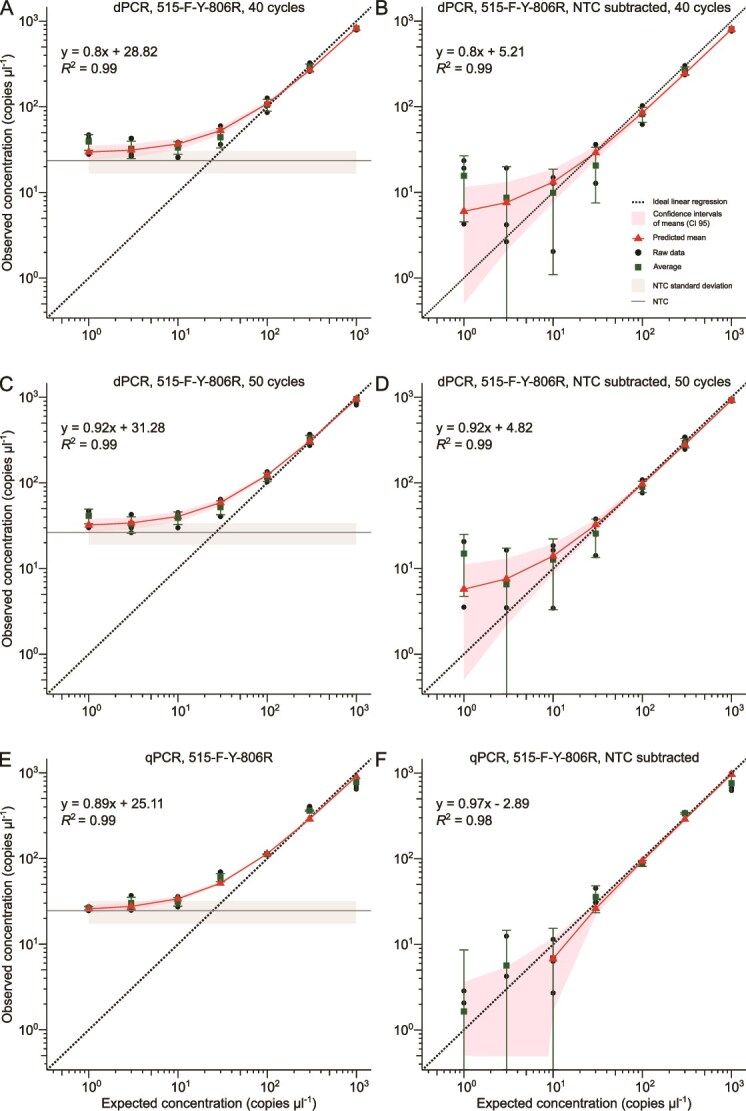
Digital PCR and qPCR performance of 515F-Y–806R primer pair. Weighted linear regression models illustrate relationship between observed and expected copy number concentration per μl of synthetic standard for dPCR after 40 PCR cycles (A), dPCR after 40 cycles with average NTCs subtracted (B), dPCR after 50 PCR cycles (C), dPCR after 50 cycles with subtracted average NTCs (D), qPCR after 40 PCR cycles (E), and qPCR after 40 PCR cycles with subtracted average NTCs (F). The diagonal solid line is the predicted mean concentration, the shadow around the solid line is 95% confidence interval of the means. Dots represent raw data and squares indicate the average concentration with standard deviation. The theoretical optimal quantification is indicated with the dashed line representing identical observed and measured concentrations. Horizontal lines represent average NTCs. Shadow around NTC lines represents standard deviation.

During dPCR testing of 16S rRNA gene primers with the synthetic standard as template, higher NTC values were observed than for P5-P7 amplification (Figs. 2 and 3, panels A and C), indicating the presence of nucleic acid contamination. Average copies for NTCs were 7.1 ± 3.7 copies μl^−1^ for 341F-518R and 23.6 ± 6.9 copies μl^−1^ for 515F-Y-806R. Furthermore, intermediate partitions were observed for 16S rRNA gene primer data that were not apparent for P5-P7 amplifications (Suppl. Figs. 4A, B, 5, and  6). These intermediate partitions may have resulted from non-specific amplification or incomplete amplicon generation, leading to truncated PCR products. To help reduce these intermediate partitions, we increased elongation time, number of cycles, annealing temperature, and adjusted primer concentrations. Increasing the elongation time had no observable effect on intermediate partition resolution (data not shown) but increasing cycle number reduced intermediate partitions for 515F-Y-806R (Suppl. Figs. 4B and 6) but not for 341F-518R amplifications (Suppl. Figs. 4A and 5). The 515F-Y-806R primer pair consistently displayed an increased number of positive partitions for NTCs, potentially due to the low annealing temperature of 50°C. Increased intermediate and positive partitions for NTCs were observed for annealing temperatures in a range from 52°C to 55°C, whereas they decreased above 55°C (data not shown). Similarly, the 341F-518R primer pair showed an increase in intermediate partitions in NTCs at temperatures below 55°C (data not shown), suggesting possible nonspecific amplification at lower annealing temperatures. For subsequent dPCR and qPCR quantifications, we used the published primer annealing temperatures to maintain their broad coverage of microbial templates.

In contrast to P5-P7 amplification results, shapes of WLR curves for 16S rRNA gene dPCR and qPCR revealed contaminant templates in NTCs, resulting in high copy number detection for low template concentrations (Figs. 2, and  3, panels A, C, and E). Additionally, high variability (40%–94% RSD for the 341F-518R, and 15%–25% for 515F-Y-806R) was observed for detection of copy numbers less than 30 copies μl^−1^, which was higher than at other concentrations (below 24% RSD, and below 15% for the 341F-518R), and the 95% CIs overlapped with those of NTCs for both primer pairs (Figs. 2 and 3, Suppl. Table 2). However, the differences in variability were not statistically significant (*P* > 0.05). When subtracting the NTC copy numbers from sample copy numbers, fitted linear curves showed more accurate results for the 341F-518R primer pair than for 515F-Y-806R (Figs. 2 and 3, panels B, D, and F). Although the fitted linear regression curves were close to ideal after NTC subtraction, the standard deviation of averages, as well as RSDs, increased for samples with copy number < 30 and < 10 copies μl^−1^ for dPCR and qPCR, respectively, for both primer pairs (Figs. 2 and 3, Suppl. Table 3). Moreover, statistical test showed significant difference in RSDs after NTC subtraction. Increasing the number of amplifications to 50 cycles led to increasing copy number estimates for NTCs to 10.7 ± 4.7 and 26.5 ± 7.4 copies μl^−1^ for dPCR with 341F-518R and 515F-Y-806R primer pairs, respectively, and had little impact on the RSD of replicates (Figs. 2 and 3, panel C, Suppl. Tables 2 and 3). The copy number detected in NTCs for qPCR were 6.5 ± 1.2 and 24.7 ± 7.2 copies μl^−1^, which were similar for dPCR results (Figs. 2 and 3, panel E). Moreover, CIs for qPCR were narrower than for dPCR for 341F-518R primer pair (Fig. 2F). Overall, linear regressions for predicting variation in measurements revealed that RSD values were negatively correlated with increasing copy number for both qPCR and dPCR (Suppl. Tables 2 and 3).

### Primer concentration

Using 2 μl of 1 × 10^4^ copies μl^−1^ synthetic standard for all reactions, we tested several primer concentrations (i.e., 100–500 nM) and cycle numbers to evaluate the effect on dPCR for both 16S rRNA gene primer pairs, with a focus on reducing intermediate partitions to improve threshold placement. Both primer pairs failed to amplify at 100 nM primer concentrations (Suppl. Fig. 7 and Suppl. Fig. 8), regardless of cycle number. The 515F-Y-806R primer pair similarly did not amplify template at 200 or 300 nM primer concentrations with 40 cycles, but some amplification was observed for 300 nM primer concentration after an additional 10 cycles. At 400 and 500 nM primer concentration, fewer intermediate partitions were observed after 50 cycles as compared to 40 cycles (Suppl. Fig. 8D-J). However, no intermediate partitions were observed for NTC amplifications with 40 or 50 cycles (Suppl. Fig. 8D-J). For the 341F-518R primer pair, the number of intermediates for 1 × 10^4^ copies μl^−1^ template and NTCs was similar (Suppl. Fig. 7).

### Template volume

**Figure 4:**
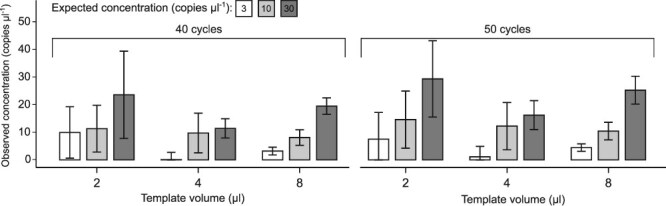
Influence of template volume on the accuracy and precision of dPCR using 515F-Y-806R primer pair. Copy number was calculated after 40 and 50 cycles of dPCR. Average NTCs were subtracted. Error bars represent the standard deviation of three replicates.

Template volumes of 2, 4, and 8 μl were tested in reactions with 3, 10, and 30 copies μl^−1^ of synthetic standard. Increasing template volume reduced quantification variability and the averaged results were closer to the expected values (Fig. 4). Pairwise comparisons between measured concentrations using 2, 4, or 8 μl template volumes were performed using the Wilcoxon rank-sum exact test. To control the false discovery rate across multiple comparisons, the Benjamini-Hochberg procedure was applied and adjusted *P* values were reported (Suppl. Table 4). The analyses showed significant differences (*P* = 0.002) among all three measured concentrations when 8 μl of template was used, with no difference observed for the lowest (2 μl) template volume (*P* > 0.05). For 4 μl template volumes, no difference was observed between 10 and 30 copies μl^−1^ (*P* = 0.48), but comparing other measured concentrations showed significant differences (*P* = 0.007).

### Inhibitors

**Figure 5:**
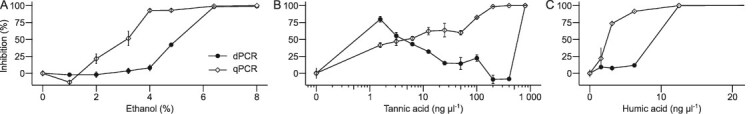
The effect of ethanol (A), tannic acid (B), and humic acid (C) on dPCR and qPCR quantification. Percent of inhibition at increasing concentration of inhibitor is shown for dPCR (solid line) and qPCR (dashed line). Error bars represent the standard deviation of three replicates.

Inhibitory effects of ethanol, humic, and tannic acids on qPCR and dPCR were tested using P5-P7 primer pair and 2 μl of the synthetic standard template at concentrations of 1 × 10^4^ copies μl^−1^. Ethanol had an inhibitory effect on qPCR with half-maximal inhibitory concentration (IC_50_) of 2.85% (± 0.28) and completely inhibited the reaction at 4% final volume (Fig. 5A). For dPCR, the IC_50_ was 4.92% (± 0.05), complete inhibition was observed at 6.5% (Fig. 5A). The shape of positive partitions changed with increasing ethanol concentration due to fluorescence intensity decreasing in middle partitions (Suppl. Fig. 9). This fluorescence intensity gradient results in positive partitions exhibiting a “V-shape” beginning at 4% ethanol, near the IC_50_ and forming a deeper V-shape as ethanol concentrations increase. At ethanol concentrations 6.5% or above, fluorescence intensities of most partitions were as low as negative partitions or no fluorescence was detected. On the corresponding fluorescence signal map of the dPCR nanoplate, black areas with no signal can be observed for higher ethanol concentrations (Suppl. Fig. 10).

**Figure 6:**
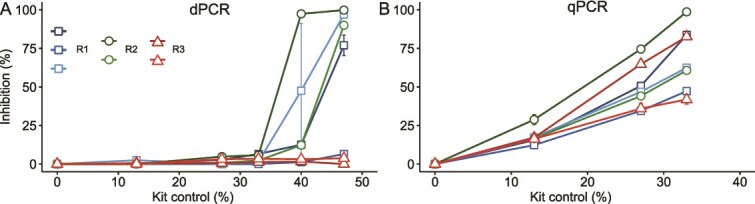
The effect of increasing volumes of kit control on dPCR (A) and qPCR (B) estimates. Percent of inhibition at increasing volume of kit control is shown for Researcher 1 (square), Researcher 2 (rhombus), and Researcher 3 (triangle). Error bars represent standard deviation.

Ethanol and other reaction inhibitors could be present in the final elution for the DNA extraction kit and inhibit PCR. Consequently, a blank extract (i.e., no sample added, “kit control”) from the PowerMax Soil DNA kit was spiked at 0–47% of the reaction volume for dPCR and 0–33% for qPCR. Inhibition in dPCR was detected for the 40% spike, whereas inhibition started at 13% for qPCR (Fig. 6). The extent of inhibition at the corresponding spike volume varied among researchers and among different batches processed by the same researcher. For dPCR, kit controls from researcher 1 exhibited 50% inhibition for a 40% spike, whereas the third kit control showed no inhibition. For researcher 2, one kit control showed complete inhibition at a 40% spike, whereas the second displayed ~10% inhibition for the same spike volume. Both kit controls isolated by researcher 3 showed no inhibition of dPCR. The partitions plot and fluorescence signal map revealed similar V-shape patterns and blacked-out areas as observed for ethanol spikes (Suppl. Figs. 11 and 12), suggesting the presence of ethanol traces in most kit controls. Variations between researchers may be due to differences in handling during DNA extraction or due to variations between kit lot numbers used. For qPCR, inhibition was observed for all kit controls, with 42% to 100% inhibition at the maximum spike volume of 33%.

Inhibition by tannic acid in dPCR did not follow a linear trend. At 1.6 ng μl^−1^, 80% inhibition was observed, but was followed by a decrease in inhibition up to 400 ng μl^−1^. Complete inhibition occurred at 800 ng μl^−1^ (Fig. 5B). Fluorescence intensity decreased with increasing tannic acid concentrations, making it difficult to distinguish between positive and negative partitions (Suppl. Fig. 13). The QIAcuity user manual states that for correct functioning of the image analysis algorithm a positive partition fluorescence intensity of 80 to 120 is needed and recommends an increase in exposure time for fluorescence below 60. The fluorescence intensity for 50 ng μl^−1^ tannic acid was ~35 and less than 20 for tannic acid concentrations above 100 ng μl^−1^. We did not increase exposure time for high tannic acid concentrations, which could have negatively affected dPCR accuracy. Additionally, numerous intermediate partitions appeared for all concentrations of tannic acid tested. Thus, even small amounts of tannic acid affected dPCR dynamics. Based on the graphical inhibition data, the IC_50_ for tannic acid was 0.5 ng μl^−1^; however, the drc package for dose–response modeling was not able to calculate using the present dataset. For qPCR, inhibition increased with tannic acid concentration, showing an IC_50_ of 10 ng μl^−1^ and complete inhibition at 200 ng μl^−1^. Tannic acid may have a stronger effect on dPCR than for qPCR, given that fluorescence intensity and copy number decreased even for 1.5 ng μl^−1^ of spike.

Digital PCR was less susceptible to humic acid inhibition than qPCR (Fig. 5C). The IC_50_ for qPCR was 2.30 ± 0.05 ng μl^−1^ and 7.45 ± 2.96 ng μl^−1^ for dPCR. Complete inhibition was observed at 12.5 ng μl^−1^ for both methods. On the partitions plot, the decrease and fluorescence intensity were seen with increasing concentration of humic acid up to 6.2 ng μl^−1^ and no positive partitions were observed for 12.5 ng μl^−1^ (Suppl. Fig. 14).

### Decontamination

**Figure 7:**
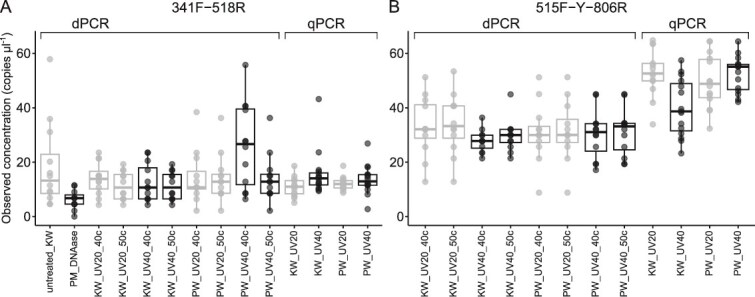
Copy numbers in NTCs after dPCR and qPCR using 341F-518R (A) or 515F-Y-806R (B) primer pair. PCR water from dPCR kit (KW) or HyPure Molecular Biology Grade Water (Cytiva) (PW) was treatment with ultraviolet light for 20 minutes (UV20) or 40 minutes (UV40). PCR was performed for either 40 (40c) or 50 (50c) cycles of PCR. Treatment of the dPCR mixture with DNAse (PM_DNAse) was also tested.

Because nucleic acid contamination of PCR reagents can negatively affect 16S rRNA gene quantification of samples, two types of PCR water were tested using 20 and 40 minutes of UV light exposure and DNAse treatment of dPCR mix was tested. The dsDNAse was not deactivated using the recommended protocol which involved a 5 min incubation at 58°C. Optimization attempts of the DNAse treatment protocol revealed that measured copy numbers were lower than expected at temperatures below 70°C, further indicating incomplete deactivation. In contrast, incubation at 75°C and 80°C led to increased number of intermediate partitions (data not shown). DTT required for DNAse inactivation negatively affected the dPCR at higher concentration (5–30 mM) and lower DTT concentration (1 mM) did not deactivate the enzyme completely at 60 and 65°C (data not shown). No significant differences were found among all treatments tested using Wilcoxon rank-sum exact test and Benjamini-Hochberg correction (Fig. 7, Suppl. Tables 5 and 6).

### Mock community and environmental samples

To further demonstrate that 16S rRNA gene dPCR is applicable to low-biomass and environmental samples, we tested the performance on groundwater, bentonite and a defined microbial community standard. The qPCR and dPCR with primer pair 515F-Y-806R yielded very similar copy number estimates for samples with ≧8969 copies μl^−1^ (mock 8968, groundwater 1, groundwater2) and both PCR type estimates varied by only 0.99- to 1.23-fold (Suppl. Fig. 15A). In comparison, the 341F-518R dPCR estimates for the same samples varied more and were increased by 1.16 for mock 8968 and decreased by 0.43 for groundwater 2. For low biomass samples with concentrations below the detection limit of the Qubit fluorometer, dPCR and qPCR varied by 1.71 to 4.71 for 341F-518R and 1.87 to 6.07 for 515F-Y-806R. Variations between copy number estimates using 40 or 50 cycles of dPCR amplification were very low for 515-Y-806R primer pair with ≦ 1.08 and higher for 341F-518R with up to 1.42-fold. On average, 341F-518R dPCR estimates were 1.24- and 1.56-fold higher than 515F-Y-806R for 40 and 50 cycles of amplification, respectively. For samples with known average genome size and 16S rRNA gene copy numbers (e.g. mock community 8968), 341F-518R dPCR varied by 0.72- and 0.63-fold to the nominal value while 515F-Y-806R only varied by 0.90 to 0.88 for 40 and 50 cycles, respectively. However, both primer pair estimates were statistically different based on a one-sample t-test (*P* < 0.05). No statistical difference to the mock 89 sample nominal value was observed for 515F-Y-806R 40 and 50 cycles dPCR values as well as the 341F-518R 40 cycle dPCR. Least variation was observed for standard 1120 for both primer pairs and cycle numbers with only 0.94- to 1.09-fold difference to the nominal value and only 515F-Y-806R 50 cycle dPCR estimates were significantly different (*P* = 0.02). As described previously for the synthetic standard, the ratio of intermediates increased for mock community and environmental samples with higher dPCR cycle numbers for 341F-518R but not for 515F-Y-806R (Suppl. Fig. 15B and C, Suppl. Figs. 16 and 17).

Average copies for NTCs in qPCR were 15.0 ± 0.8 copies μl^−1^ for 341F-518R and 14.1 ± 1.2 copies μl^−1^ for 515F-Y-806R. NTC with 20.0 ± 6.0 and 27.78 ± 8.2 copies μl^−1^ were detected in 341F-518R 40 and 50 cycle dPCR, respectively, and 34.9 ± 12.0 and 34.03 ± 14.3 copies μl^−1^ were detected in 515F-Y-806R 40 and 50 cycle dPCR.

## Discussion

Quantitative PCR is widely used in microbial ecology research to determine gene copy numbers of environmental samples using group specific or pan-domain targets. In this study we compared qPCR and dPCR with two commonly used primer pairs, 341F-518R and 515F-Y-806R, targeting the 16S rRNA gene, with emphasis on low biomass bacterial and prokaryotic quantification, respectively. Adjusting dPCR conditions can improve the efficiency of reaction and help with resolution of positive and negative partitions [52, 53]. We found that increasing the cycle number from 40 to 50 cycles decreased intermediate partitions with high concentration synthetic standard templates for 300 to 500 nM 515F-Y-806R primer pair. The decrease of intermediate partitions with 50 cycles of dPCR and 400 nM 515F-Y-806R primer pair was also observed when tested on a defined mock community and environmental samples. Furthermore, copy number detection was unchanged with 40 and 50 cycles of dPCR when using 515F-Y-806R but an increase in intermediate partitions and copy number detection was observed when using 341F-518R with additional 10 cycles. Combining 400 to 500 nM primer concentration with an additional 10 thermocycles yielded a relatively low number of intermediate partitions for high-concentration samples for both primer pairs. Although increasing annealing temperatures yielded lower numbers of intermediate partitions for both primer pairs, we maintained the published temperatures in our study to ensure their universal coverage. Both primer pairs were designed for broad bacterial or prokaryotic amplification, which requires degenerate bases and relaxed annealing temperatures. This design requirement for a universal primer does not necessarily yield a primer pair most suitable for qPCR and dPCR. More specific primer and optimized annealing temperatures may be preferable for quantitative PCRs, despite the convenience of one-step pan-domain quantification.

In our research, SYBR Green and EvaGreen based assays were employed for qPCR and dPCR respectively. However, TaqMan (primer/probe) based quantifications are thought to be more sensitive and more specific, avoiding non-specific amplification [7]. In our experience, 16S rRNA gene probe based dPCR with UNI340F-UNI806R primer pair and TM516F TaqMan FAM-labeled probe [54] did not show higher sensitivity when using QIAcuity Probe PCR Kit (Qiagen, cat no. 250102) or UCP Probe PCR Kit (Qiagen, cat no. 250121). Similar non-specific amplification and detection limits were observed when compared to the EvaGreen-based dPCR assays (data not shown). Further comparison between these two methods using different primer/probe pairs would be needed to fully compare performances.

Quantification of the 16S rRNA gene was accurate for low-biomass synthetic standard samples down to 30 copies μl^−1^ concentration range. Previous research suggested that dPCR offers more sensitive detection while qPCR has a much broader dynamic detection range [15]. Our results showed that dPCR was more precise when using the P5-P7 primer pair, but differences were less pronounced when using the 16S rRNA gene specific primers, likely due to the presence of nucleic acid contamination (“kitome”) that is not present for the P5-P7 assays. Previous research showed that DNA degradation can negatively affect 16S rRNA gene copy number quantification [55] and assessment of DNA quality was recommended. Although accurate quantification of the synthetic standard appeared to be predominantly affected by reagent contamination, shearing of environmental samples during DNA extraction and handling might affect precision.

A previous study demonstrated that bacterial DNA contamination in extraction kits and laboratory reagents can significantly impact the outcomes of microbiome studies, especially when analyzing samples with low microbial biomass [26]. In our study, putative contamination accounted for ~10 copies μl^−1^ and ~ 25 copies μl^−1^ of 16S rRNA genes in NTCs for 341F–518R and 515F-Y–806R, respectively, for both dPCR and qPCR. Several methods reduce contamination in reagents, including UV irradiation and DNAse treatment, which showed varying success in previous studies [27, 56–58]. In this study, UV treatment of PCR water and DNAse treatment on PCR mixtures did not reduce contaminant copy number detection in NTCs. Higher concentrations of DTT negatively affected the dPCR and lower DTT concentrations in combination with temperatures below 70°C, did not deactivate the DNAse enzyme completely. Although DNAse treatment reduced contaminating microbial DNA from PCR reagents, negative effects on PCR performance and incomplete DNAse inhibition was previously reported, affecting bacterial richness in sequencing studies or PCR performance [59–62]. Further optimization may be required. Subtraction of NTCs from sample data improved accuracy of quantification but high variability was observed for samples containing 30 copies μl^−1^ or less. The broader amplicon target range of 515F-Y–806R likely leads to enhanced contaminant detection and reduced sensitivity compared to 341F-518R and thus the latter may be the preferred primer pair.

Adding larger template volumes can improve precision and limit of detection for dPCR and qPCR. However, the presence of inhibitors such as ethanol and humic acids in DNA extracts negatively affect PCR and can lead to inhibition and template underestimation. Inhibition by humic acid has been reported for soil studies because its co-extraction is a concern in the PCR amplification of soil DNA [63]. Research shows that humic substances are not effectively removed in soil DNA extracts using spin columns and can contain 0.01–59 ng of humic acid per gram of soil [64, 65]. A previous study demonstrated that different commercial qPCR kits exhibit varying levels of inhibition resistance [66]. Furthermore, several kits were more sensitive to inhibitors present in environmental samples. Others have reported that dPCR is less sensitive to PCR inhibitors co-extracted with DNA as compared to qPCR [24, 67–69]. Most studies regarding inhibition were performed using probes and digital droplet PCR. In this study, we showed that dPCR was less sensitive to ethanol and humic acid than qPCR but likely more sensitive to tannic acid. It was previously shown that humic acids interact with the template DNA and the polymerase, subsequently preventing the enzymatic reaction even at low concentrations and may quench fluorescence of double-stranded DNA-binding dyes (SYBR and Eva Green) [70, 71]. Tannic acid may bind magnesium, a co-factor critical for DNA polymerases, thereby inhibiting polymerase activity [72]. However, the reason for the increase in copy number for dPCR at higher concentrations is unknown.

Ethanol is a component in the washing solution of DNA isolation kits, and previous studies have shown that ethanol traces can remain in the final DNA elution, potentially affecting PCR [73, 74]. The mechanism of inhibition by ethanol is poorly understood but alcohols may precipitate DNA and stop the reaction [75]. Inhibition was also observed during spiking experiments with kit control, suggesting the presence of ethanol residues in the DNA extract. The amount of ethanol traces may vary depending on the DNA extraction batch or minor inconsistencies during handling, such as the duration or efficiency of ethanol removal in the final steps of the extraction protocol. Additionally, we eluted the DNA in 2 ml of elution buffer, instead of the 5 ml recommended by the DNeasy PowerMax Soil Kit Handbook, to achieve a more concentrated DNA solution. However, this may aggravate the inhibition effect caused by residual ethanol. Although we attempted to extend the evaporation time before the elution step to reduce ethanol carryover, this adjustment did not mitigate the issue (data not shown). Alternative DNA concentration and purification methods may be tested if needed to minimize ethanol residues in DNA samples. Besides, 26k nanoplates allow the use of the high sample volumes (e.g. 8 μl) for higher precision with reduced dPCR inhibition potential because the total reaction volume is 3.3-fold higher than the 8.5k plates.

## Conclusion

We demonstrated the reliability of chip-based dPCR quantification using commonly used 16S rRNA gene primer pairs for application of low-biomass samples. Despite the advantages of this method in precise low copy number estimation, we observed variability in very low-biomass synthetic standard samples with less than 30 copies μl^−1^. To achieve more accurate results and higher confidence, technical replicates (≥2) should be employed for low biomass samples. Higher template volumes (>4 μl) will improve dPCR precision, but inhibition tests should also be performed. Positive partitions should be investigated in 1D scatter plots provided by the QIAcuity Software Suite as inhibition may be evident by V-shapes of positive partitions or overall reduced fluorescence signals. Additionally, the tested 16S rRNA gene primers detected microbial DNA contamination for both dPCR and qPCR mixtures, decreasing the sensitivity for both methods. Subtracting kit control signal can help eliminate the contribution of contaminants introduced during DNA extraction and dPCR preparation. Even though dPCR was found to be less susceptible to inhibition by ethanol and humic acid than qPCR, assessment of potential inhibition should be conducted prior to any analysis of sample template concentrations.

## Supplementary Material

Koziaevams_supp_clean_ycaf115

## Data Availability

All data generated or analysed during this study are included in this published article or in the Supplementary Information files.
