# Supplementary material for: Validating digital polymerase chain reaction for 16S rRNA gene amplification from low biomass environmental samples
Source: ISME Commun. 2025 Jul 9;5(1):ycaf115. doi: 10.1093/ismeco/ycaf115 (PMC12342375; doi:10.1093/ismeco/ycaf115)
Supplement: Koziaevams_supp_clean_ycaf115 [file koziaevams_supp_clean_ycaf115.docx]

**Validating digital PCR for 16S rRNA gene amplification from low biomass environmental samples.**

V.V. Koziaeva, K. Engel, J. D. Neufeld

Department of Biology, University of Waterloo, Waterloo, Ontario, Canada

*Supplementary materials*

Suppl. Table 1. Relative standard deviation for P5-P7 primer pair tested at different synthetic standard dilutions

| Dilution | Relative standard deviation (RSD), % | |
| --- | --- | --- |
|  | Unsubtracted NTC | Subtracted NTC |
| 1 | 200 | 10857 |
| 10 | 16 | 17 |
| 100 | 12 | 12 |
| 1000 | 7 | 7 |
| 10000 | 6 | 6 |
| NTC | 200 | NA^1^ |
| Statistics | | |
| Regression slope | -1.73 | -85.84 |
| Regression *R*^2^ | 0.966 | 0.98 |
| Equality RSD *p*-value | 1.88E-06 | 5.31E-09 |
| ^1^ NA – not applicable | | |

Suppl. Table 2. Relative standard deviation for each 16S rRNA primer pair tested at different synthetic standard dilutions. No subtraction of NTCs was performed

| Dilution | Relative standard deviation (RSD), % | | | | | |
| --- | --- | --- | --- | --- | --- | --- |
|  | dPCR | | | | qPCR | |
|  | 341F-518R | | 515F-Y-806R | | 341F-518R | 515F-Y-806R |
|  | 40 cycles | 50 cycles | 40 cycles | 50 cycles |  |  |
| 1 | 93 | 81 | 26 | 24 | 18 | 6 |
| 3 | 52 | 65 | 28 | 26 | 87 | 20 |
| 10 | 23 | 18 | 21 | 21 | 10 | 14 |
| 30 | 49 | 39 | 31 | 23 | 5 | 13 |
| 100 | 23 | 23 | 19 | 14 | 34 | 1 |
| 300 | 19 | 18 | 12 | 15 | 5 | 7 |
| 1 000 | 11 | 11 | 4 | 3 | 2 | 26 |
| NTC | 17 | 20 | 9 | 10 | 18 | 29 |
| Statistics | | | | | | |
| Regression slope | -0.457 | -0.454 | -0.044 | -0.056 | -0.089 | -0.023 |
| Regression *R*^2^ | 0.686 | 0.66 | 0.311 | 0.678 | 0.189 | 0.297 |
| Equality RSD *p*-value | 0.230 | 0.198 | 0.433 | 0.391 | 0.000 | 0.072 |

Suppl. Table 3. Relative standard deviation for each 16S rRNA primer pair tested at different synthetic standard dilutions. Subtraction of NTCs was performed

| Dilution | Relative standard deviation (RSD), % | | | | | |
| --- | --- | --- | --- | --- | --- | --- |
|  | dPCR | | | | qPCR | |
|  | 341F-518R | | 515F-Y-806R | | 341F-518R | 515F-Y-806R |
|  | 40 cycles | 50 cycles | 40 cycles | 50 cycles |  |  |
| 1 | 402 | 1139 | 64 | 66 | 1341 | 89 |
| 3 | 109 | 296 | 105 | 133 | 610 | 109 |
| 10 | 46 | 42 | 70 | 64 | 16 | 64 |
| 30 | 61 | 53 | 66 | 46 | 7 | 23 |
| 100 | 26 | 26 | 25 | 18 | 36 | 2 |
| 300 | 19 | 18 | 13 | 16 | 5 | 2 |
| 1 000 | 11 | 11 | 4 | 3 | 2 | 27 |
| Statistics | | | | | | |
| Regression slope | -2.145 | -2.145 | -0.22 | -0.345 | -9.564 | -0.439 |
| Regression *R*^2^ | 0.664 | 0.664 | 0.382 | 0.419 | 0.796 | 0.968 |
| Equality RSD *p*-value | 0.058 | 0.016 | 0.050 | 0.021 | 0.000 | 0.001 |

Suppl. Table 4. Wilcoxon rank-sum test with Benjamini-Hochberg *p* value adjustments for template volume analysis.

| Template volume, µl | 2 | | 4 | | 8 | |
| --- | --- | --- | --- | --- | --- | --- |
| Concentration, cp µl^-1^ | 3 | 10 | 3 | 10 | 3 | 10 |
| 10 | 0.82 | - | 0.0065 | - | 0.0022 | - |
| 30 | 0.19 | 0.36 | 0.0065 | 0.4848 | 0.0022 | 0.0022 |

Suppl. Table 5. Wilcoxon rank-sum test with Benjamini-Hochberg *p* value adjustments for decontamination treatment analysis for 341F-518R primer pair.

|  | group1 | group2 | n1 | n2 | statistic | *P* | *P*.adj | *P*.adj.signif |
| --- | --- | --- | --- | --- | --- | --- | --- | --- |
| dPCR | KW_UV20_40c | KW_UV20_50c | 12 | 12 | 88 | 0.371 | 0.797 | ns^1^ |
| dPCR | KW_UV20_40c | KW_UV40_40c | 12 | 12 | 83 | 0.544 | 0.848 | ns |
| dPCR | KW_UV20_40c | KW_UV40_50c | 12 | 12 | 88 | 0.371 | 0.797 | ns |
| dPCR | KW_UV20_40c | PM_DNAase | 12 | 12 | 112 | 0.022 | 0.169 | ns |
| dPCR | KW_UV20_40c | PW_UV20_40c | 12 | 12 | 79 | 0.707 | 0.909 | ns |
| dPCR | KW_UV20_40c | PW_UV20_50c | 12 | 12 | 76 | 0.84 | 0.948 | ns |
| dPCR | KW_UV20_40c | PW_UV40_40c | 12 | 12 | 38.5 | 0.057 | 0.255 | ns |
| dPCR | KW_UV20_40c | PW_UV40_50c | 12 | 12 | 76 | 0.84 | 0.948 | ns |
| dPCR | KW_UV20_40c | untreated_KW | 12 | 12 | 70 | 0.931 | 0.998 | ns |
| dPCR | KW_UV20_50c | KW_UV40_40c | 12 | 12 | 65 | 0.707 | 0.909 | ns |
| dPCR | KW_UV20_50c | KW_UV40_50c | 12 | 12 | 72 | 1 | 1 | ns |
| dPCR | KW_UV20_50c | PM_DNAase | 12 | 12 | 99 | 0.128 | 0.384 | ns |
| dPCR | KW_UV20_50c | PW_UV20_40c | 12 | 12 | 62.5 | 0.603 | 0.848 | ns |
| dPCR | KW_UV20_50c | PW_UV20_50c | 12 | 12 | 58 | 0.443 | 0.797 | ns |
| dPCR | KW_UV20_50c | PW_UV40_40c | 12 | 12 | 32 | 0.02 | 0.169 | ns |
| dPCR | KW_UV20_50c | PW_UV40_50c | 12 | 12 | 58 | 0.443 | 0.797 | ns |
| dPCR | KW_UV20_50c | untreated_KW | 12 | 12 | 56 | 0.378 | 0.797 | ns |
| dPCR | KW_UV40_40c | KW_UV40_50c | 12 | 12 | 79 | 0.707 | 0.909 | ns |
| dPCR | KW_UV40_40c | PM_DNAase | 12 | 12 | 98 | 0.143 | 0.402 | ns |
| dPCR | KW_UV40_40c | PW_UV20_40c | 12 | 12 | 62.5 | 0.603 | 0.848 | ns |
| dPCR | KW_UV40_40c | PW_UV20_50c | 12 | 12 | 62.5 | 0.603 | 0.848 | ns |
| dPCR | KW_UV40_40c | PW_UV40_40c | 12 | 12 | 35 | 0.033 | 0.187 | ns |
| dPCR | KW_UV40_40c | PW_UV40_50c | 12 | 12 | 62.5 | 0.603 | 0.848 | ns |
| dPCR | KW_UV40_40c | untreated_KW | 12 | 12 | 60 | 0.514 | 0.848 | ns |
| dPCR | KW_UV40_50c | PM_DNAase | 12 | 12 | 99 | 0.128 | 0.384 | ns |
| dPCR | KW_UV40_50c | PW_UV20_40c | 12 | 12 | 62.5 | 0.603 | 0.848 | ns |
| dPCR | KW_UV40_50c | PW_UV20_50c | 12 | 12 | 58 | 0.443 | 0.797 | ns |
| dPCR | KW_UV40_50c | PW_UV40_40c | 12 | 12 | 32 | 0.02 | 0.169 | ns |
| dPCR | KW_UV40_50c | PW_UV40_50c | 12 | 12 | 58 | 0.443 | 0.797 | ns |
| dPCR | KW_UV40_50c | untreated_KW | 12 | 12 | 56 | 0.378 | 0.797 | ns |
| dPCR | PM_DNAase | PW_UV20_40c | 12 | 12 | 38 | 0.052 | 0.255 | ns |
| dPCR | PM_DNAase | PW_UV20_50c | 12 | 12 | 32 | 0.02 | 0.169 | ns |
| dPCR | PM_DNAase | PW_UV40_40c | 12 | 12 | 24 | 0.005 | 0.169 | ns |
| dPCR | PM_DNAase | PW_UV40_50c | 12 | 12 | 32 | 0.02 | 0.169 | ns |
| dPCR | PM_DNAase | untreated_KW | 12 | 12 | 34.5 | 0.032 | 0.187 | ns |
| dPCR | PW_UV20_40c | PW_UV20_50c | 12 | 12 | 70.5 | 0.954 | 0.998 | ns |
| dPCR | PW_UV20_40c | PW_UV40_40c | 12 | 12 | 40.5 | 0.073 | 0.288 | ns |
| dPCR | PW_UV20_40c | PW_UV40_50c | 12 | 12 | 70.5 | 0.954 | 0.998 | ns |
| dPCR | PW_UV20_40c | untreated_KW | 12 | 12 | 67 | 0.799 | 0.948 | ns |
| dPCR | PW_UV20_50c | PW_UV40_40c | 12 | 12 | 41.5 | 0.083 | 0.288 | ns |
| dPCR | PW_UV20_50c | PW_UV40_50c | 12 | 12 | 72 | 1 | 1 | ns |
| dPCR | PW_UV20_50c | untreated_KW | 12 | 12 | 68 | 0.843 | 0.948 | ns |
| dPCR | PW_UV40_40c | PW_UV40_50c | 12 | 12 | 102.5 | 0.083 | 0.288 | ns |
| dPCR | PW_UV40_40c | untreated_KW | 12 | 12 | 93 | 0.242 | 0.641 | ns |
| dPCR | PW_UV40_50c | untreated_KW | 12 | 12 | 68 | 0.843 | 0.948 | ns |
| qPCR | KW_UV20 | KW_UV40 | 16 | 16 | 66 | 0.019 | 0.113 | ns |
| qPCR | KW_UV20 | PW_UV20 | 16 | 16 | 92 | 0.184 | 0.276 | ns |
| qPCR | KW_UV20 | PW_UV40 | 16 | 16 | 77 | 0.056 | 0.169 | ns |
| qPCR | KW_UV40 | PW_UV20 | 16 | 16 | 173 | 0.094 | 0.188 | ns |
| qPCR | KW_UV40 | PW_UV40 | 16 | 16 | 139 | 0.696 | 0.696 | ns |
| qPCR | PW_UV20 | PW_UV40 | 16 | 16 | 99 | 0.287 | 0.344 | ns |
| ^1^ ns – not significant | | | | | | | | |

Suppl. Table 6. Wilcoxon rank-sum test with Benjamini-Hochberg *p* value adjustments for decontamination treatment analysis for 515F-Y-806R primer pair.

|  | group1 | group2 | n1 | n2 | statistic | *p* | *p*.adj | *p*.adj.signif |
| --- | --- | --- | --- | --- | --- | --- | --- | --- |
| dPCR | KW_UV20_40c | KW_UV20_50c | 12 | 12 | 65.5 | 0.729 | 0.955 | ns^1^ |
| dPCR | KW_UV20_40c | KW_UV40_40c | 12 | 12 | 102 | 0.088 | 0.892 | ns |
| dPCR | KW_UV20_40c | KW_UV40_50c | 12 | 12 | 90 | 0.312 | 0.892 | ns |
| dPCR | KW_UV20_40c | PW_UV20_40c | 12 | 12 | 85 | 0.478 | 0.892 | ns |
| dPCR | KW_UV20_40c | PW_UV20_50c | 12 | 12 | 79 | 0.713 | 0.955 | ns |
| dPCR | KW_UV20_40c | PW_UV40_40c | 12 | 12 | 79.5 | 0.686 | 0.955 | ns |
| dPCR | KW_UV20_40c | PW_UV40_50c | 12 | 12 | 72 | 1 | 1 | ns |
| dPCR | KW_UV20_50c | KW_UV40_40c | 12 | 12 | 104 | 0.069 | 0.892 | ns |
| dPCR | KW_UV20_50c | KW_UV40_50c | 12 | 12 | 94 | 0.214 | 0.892 | ns |
| dPCR | KW_UV20_50c | PW_UV20_40c | 12 | 12 | 87.5 | 0.386 | 0.892 | ns |
| dPCR | KW_UV20_50c | PW_UV20_50c | 12 | 12 | 85 | 0.478 | 0.892 | ns |
| dPCR | KW_UV20_50c | PW_UV40_40c | 12 | 12 | 88 | 0.378 | 0.892 | ns |
| dPCR | KW_UV20_50c | PW_UV40_50c | 12 | 12 | 82.5 | 0.564 | 0.929 | ns |
| dPCR | KW_UV40_40c | KW_UV40_50c | 12 | 12 | 52 | 0.26 | 0.892 | ns |
| dPCR | KW_UV40_40c | PW_UV20_40c | 12 | 12 | 53 | 0.285 | 0.892 | ns |
| dPCR | KW_UV40_40c | PW_UV20_50c | 12 | 12 | 49 | 0.193 | 0.892 | ns |
| dPCR | KW_UV40_40c | PW_UV40_40c | 12 | 12 | 55 | 0.341 | 0.892 | ns |
| dPCR | KW_UV40_40c | PW_UV40_50c | 12 | 12 | 53 | 0.285 | 0.892 | ns |
| dPCR | KW_UV40_50c | PW_UV20_40c | 12 | 12 | 66 | 0.751 | 0.955 | ns |
| dPCR | KW_UV40_50c | PW_UV20_50c | 12 | 12 | 61.5 | 0.564 | 0.929 | ns |
| dPCR | KW_UV40_50c | PW_UV40_40c | 12 | 12 | 64.5 | 0.686 | 0.955 | ns |
| dPCR | KW_UV40_50c | PW_UV40_50c | 12 | 12 | 58 | 0.436 | 0.892 | ns |
| dPCR | PW_UV20_40c | PW_UV20_50c | 12 | 12 | 67.5 | 0.817 | 0.955 | ns |
| dPCR | PW_UV20_40c | PW_UV40_40c | 12 | 12 | 73 | 0.977 | 1 | ns |
| dPCR | PW_UV20_40c | PW_UV40_50c | 12 | 12 | 68 | 0.843 | 0.955 | ns |
| dPCR | PW_UV20_50c | PW_UV40_40c | 12 | 12 | 75 | 0.887 | 0.955 | ns |
| dPCR | PW_UV20_50c | PW_UV40_50c | 12 | 12 | 69 | 0.887 | 0.955 | ns |
| dPCR | PW_UV40_40c | PW_UV40_50c | 12 | 12 | 59 | 0.478 | 0.892 | ns |
| qPCR | KW_UV20 | KW_UV40 | 16 | 16 | 204 | 0.003 | 0.013 | *^2^ |
| qPCR | KW_UV20 | PW_UV20 | 16 | 16 | 157 | 0.287 | 0.43 | ns |
| qPCR | KW_UV20 | PW_UV40 | 16 | 16 | 146 | 0.515 | 0.618 | ns |
| qPCR | KW_UV40 | PW_UV20 | 16 | 16 | 72 | 0.035 | 0.07 | ns |
| qPCR | KW_UV40 | PW_UV40 | 16 | 16 | 54 | 0.004 | 0.013 | * |
| qPCR | PW_UV20 | PW_UV40 | 16 | 16 | 121 | 0.809 | 0.809 | ns |
| ^1^ ns – not significant  ^2^ * - 0.01≤*p*<0.05 | | | | | | | | |


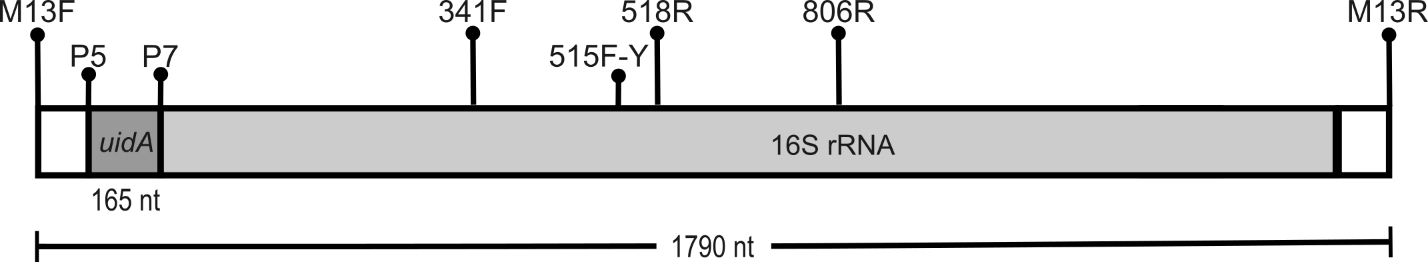


Suppl. Fig.1. Map of the synthetic DNA standard fragment showing location of primer sites used in this research.


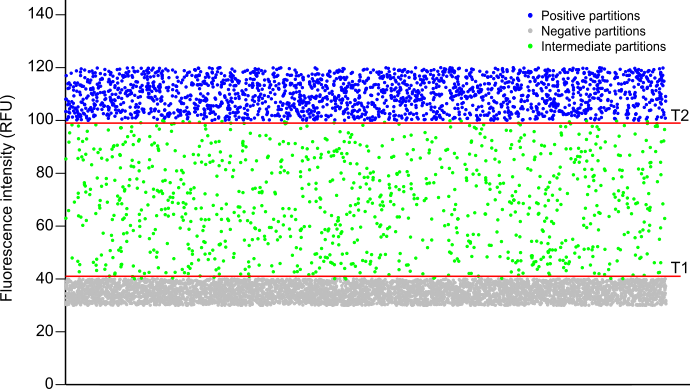


Suppl. Fig 2. Map illustrating determination of positive, negative, and intermediate partitions. Red lines (T1 and T2) represent high and low thresholds for calculating the number of intermediates.


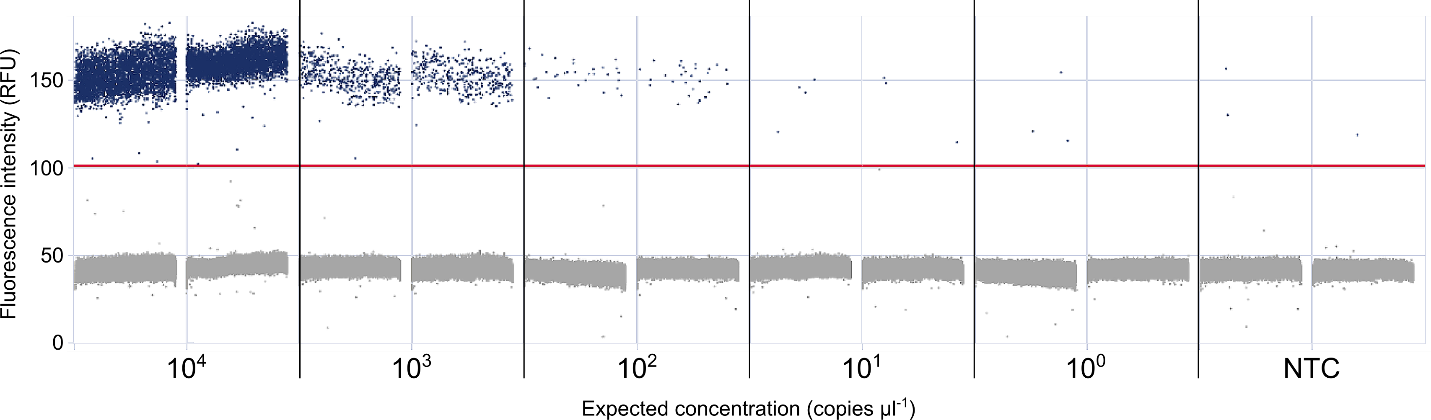


Suppl. Fig. 3. Distribution of positive (blue dots) and negative (grey dots) partitions for P5-P7 primer pair at different template concentrations (copies µl^-1^ synthetic standard added to reaction). Red line represents the threshold between positive and negative partitions used by QIAcuity software for copy number calculations. Duplicate reactions are shown.





Suppl. Fig. 4. Effect of primer concentration on the ratio of intermediate to positive partitions for 341F-518R (A) and 515F-Y-806R (B) primer pairs after 40 and 50 cycles of dPCR. Ratio of intermediates are shown without template addition (left) or with 10 000 cp/µl of template (right). For low primer concentration no positive partitions were observed and ratios of intermediates could not be calculated (NA). Panel C shows the effect of template concentration on the ratio of intermediate to positive partitions for P5-P7 after 40 cycles dPCR. Error bars represent standard deviation of two replicates.


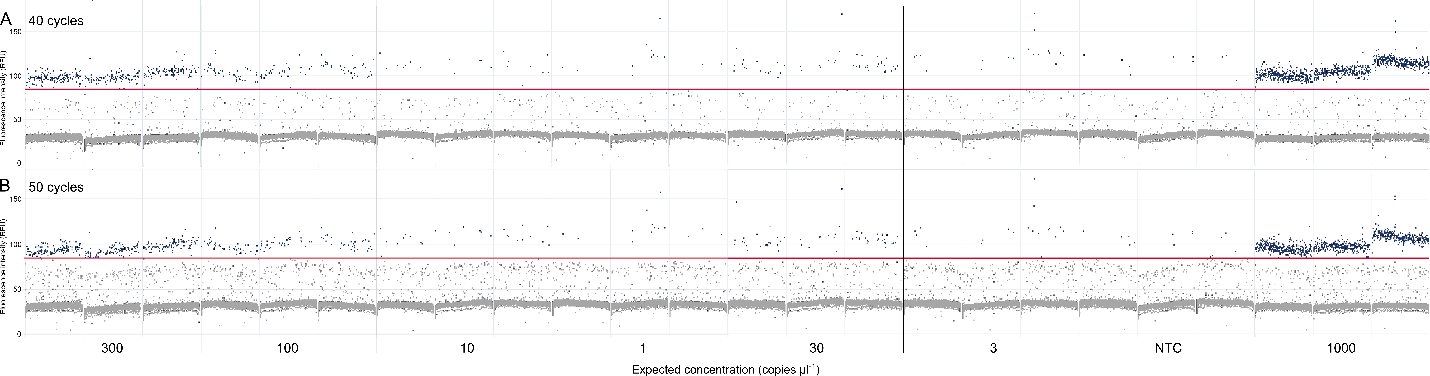


Suppl. Fig. 5 Distribution of positive (blue dots) and negative (grey dots) partitions for 341F-518R primer pair at different template concentrations (copies µl^-1^ synthetic standard added to reaction) using 40 (A) or 50 (B) PCR cycles. Red line represents the threshold between positive and negative partitions used by QIAcuity software for copy number calculations. Triplicate reactions are shown.


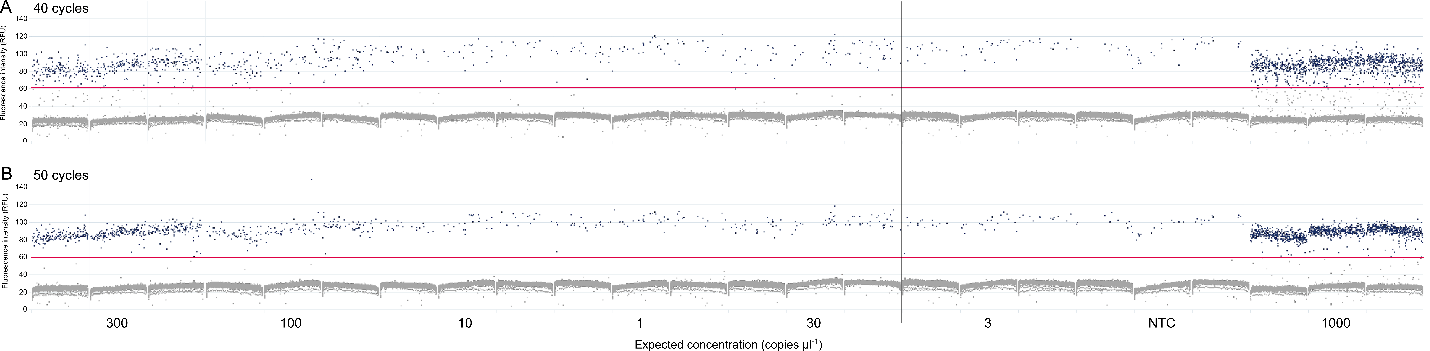


Suppl. Fig. 6 Distribution of positive (blue dots) and negative (grey dots) partitions for 515F-Y-806R primer pair at different template concentrations (copies µl^-1^ synthetic standard added to reaction) using 40 (A)or 50 (B) PCR cycles. Red line represents the threshold between positive and negative partitions.


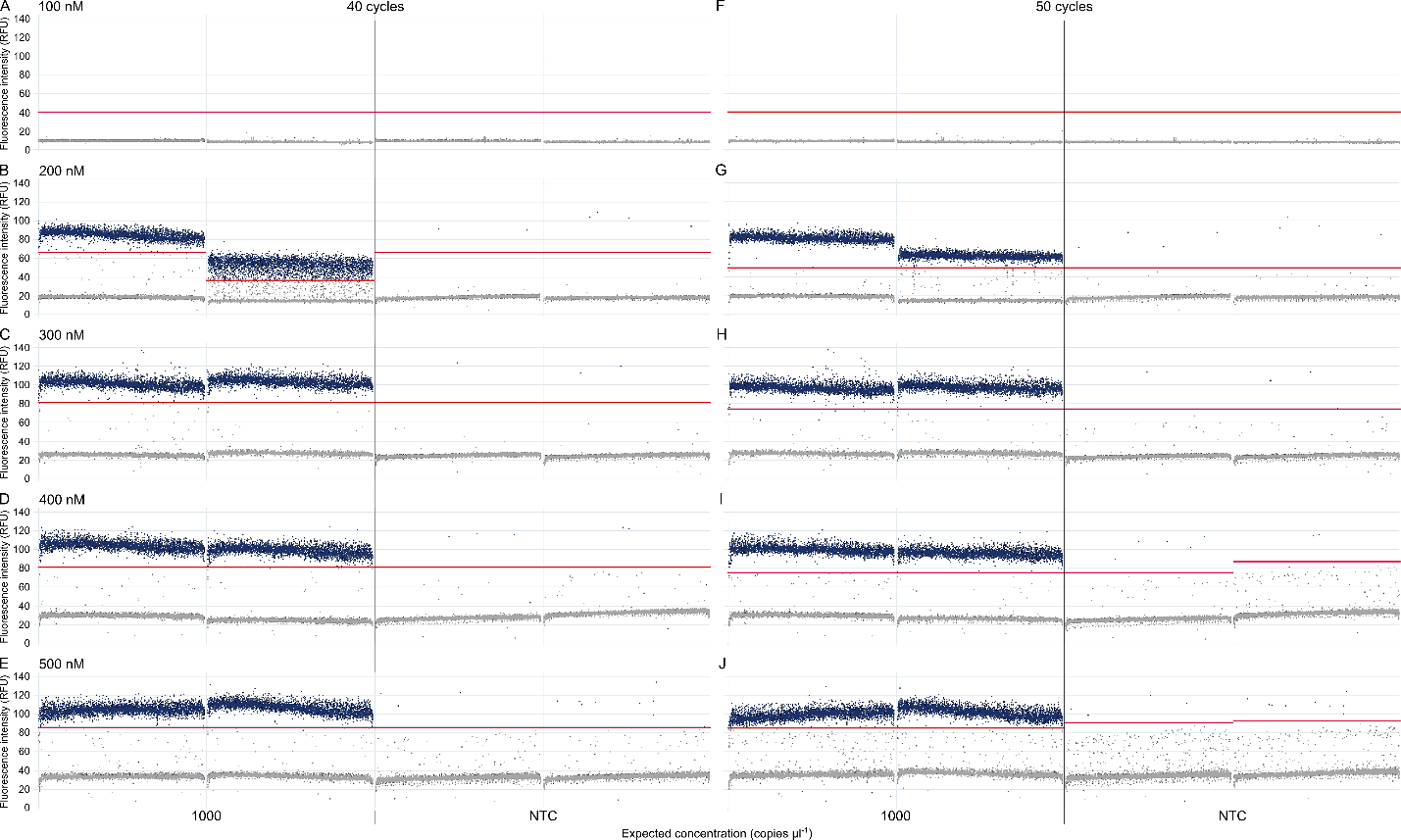


Suppl. Fig. 7. Distribution of positive (blue dots) and negative (grey dots) partitions for 341F-518R primer pair at different primer and template concentrations. A – E) 40 PCR cycles with 100 – 500 nM of primers, F – J) – 50 PCR cycles with 100 – 500 nM of primers. Red line represents the threshold between positive and negative partitions.


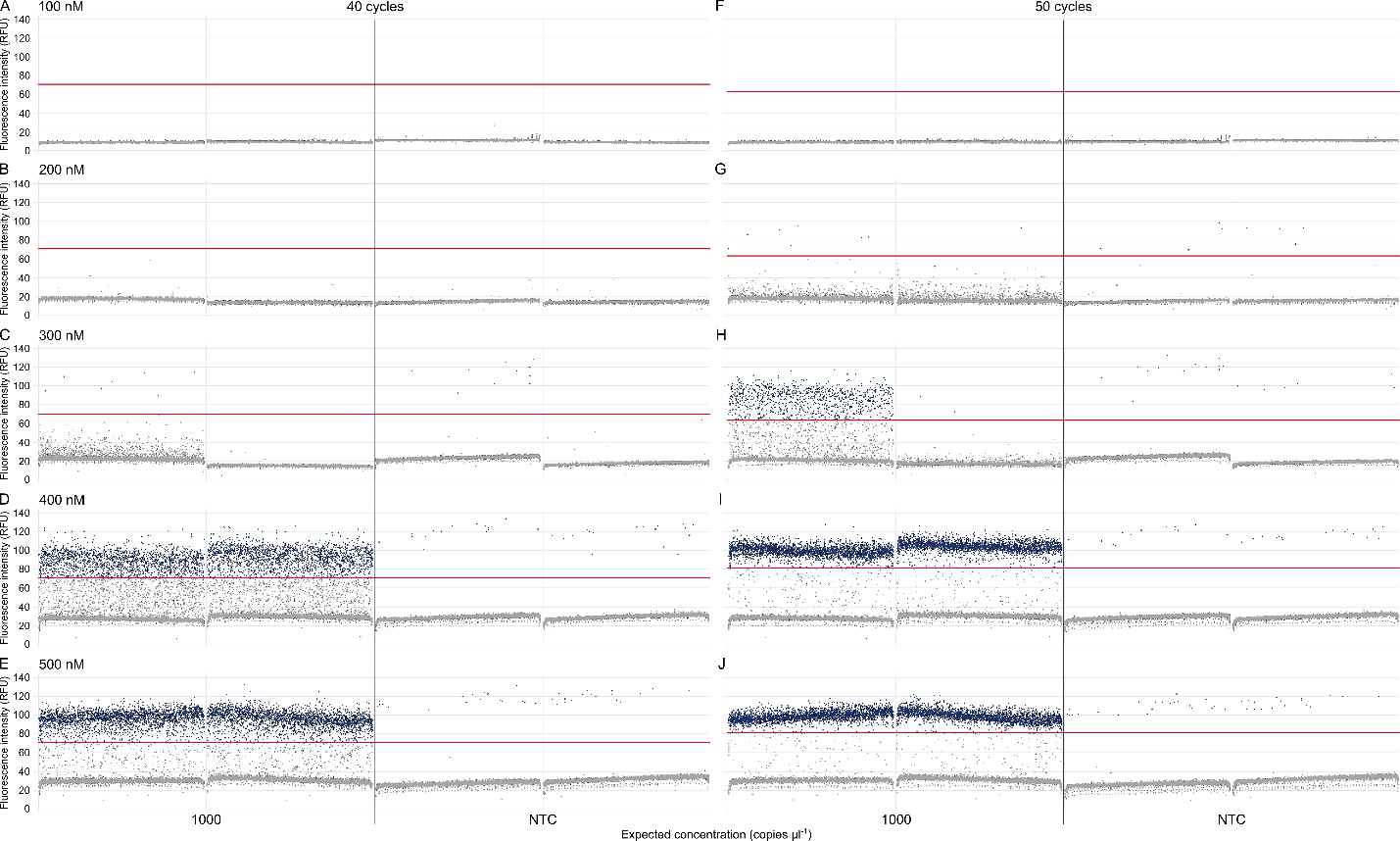


Suppl. Fig. 8. Distribution of positive (blue dots) and negative (grey dots) partitions for 515F-Y – 806R primer pair at different primer and template concentrations. A – E) 40 PCR cycles with 100 – 500 nM of primers, F – J) – 50 PCR cycles with 100 – 500 nM of primers. Red line represents the threshold between positive and negative partitions.


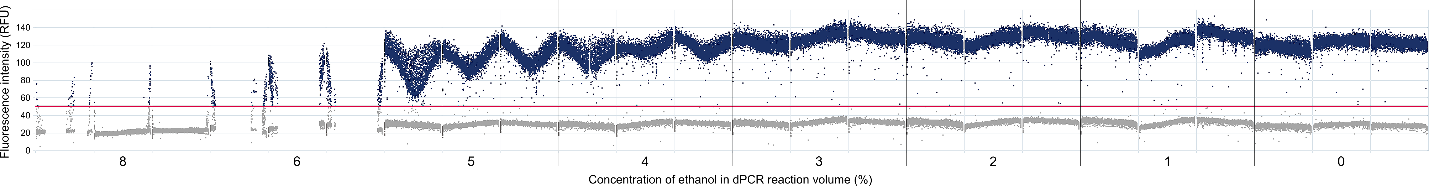


Suppl. Fig. 9. Effect of ethanol on distribution of positive (blue dots) partitions. Red line represents the threshold between positive and negative partitions. Triplicate reactions are shown.


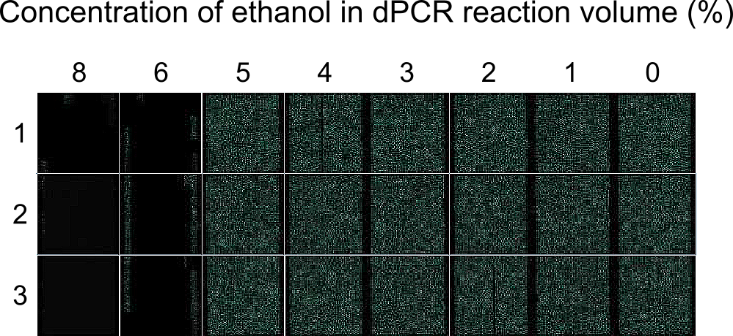


Suppl. Fig. 10. Fluorescence signal map of wells with different concentrations of ethanol. Triplicate reactions are shown (numbers on the left).


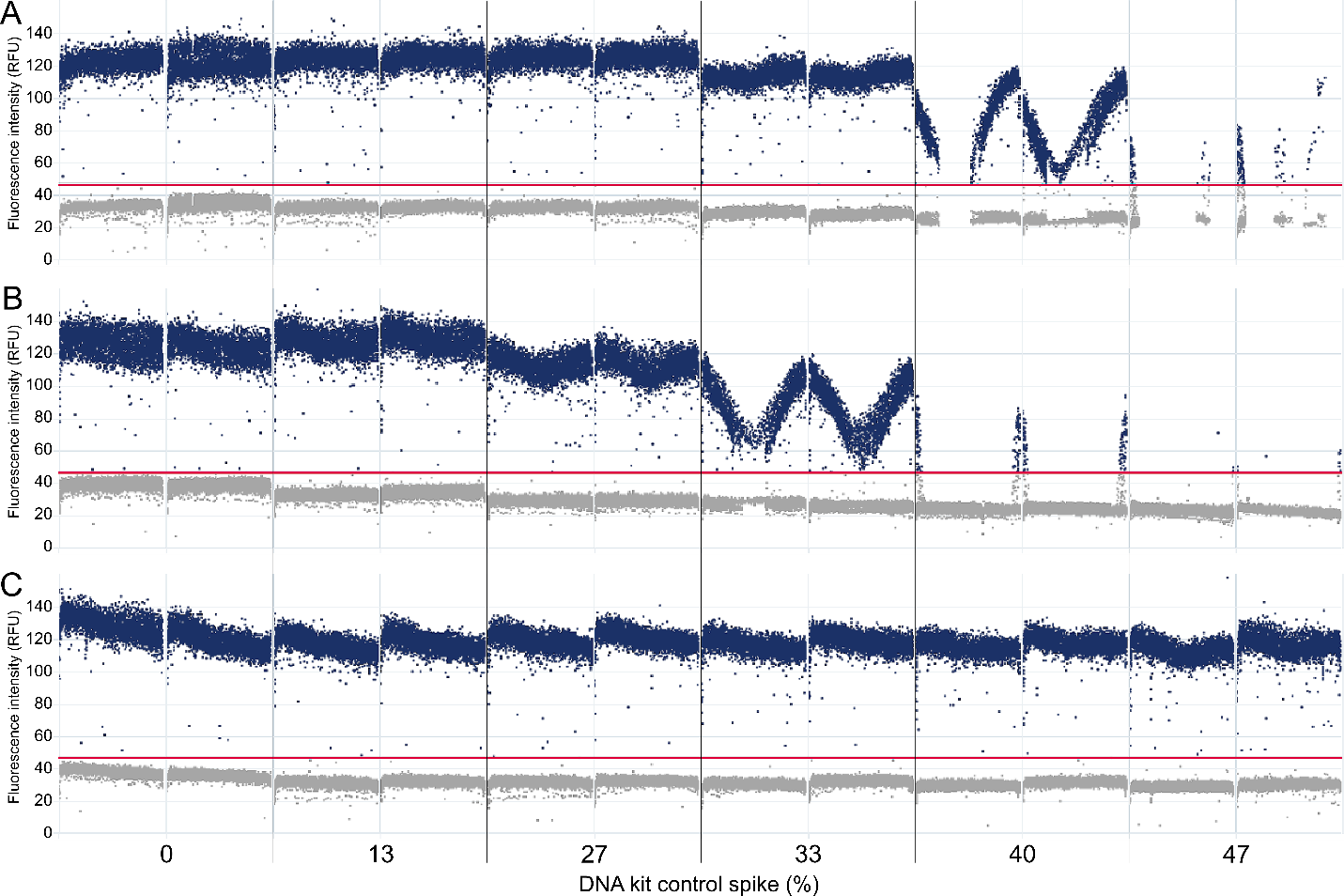


Suppl. Fig. 11. Effect of kit control spike volume on distribution of positive (blue dots) partitions. DNA extraction kit controls were obtained by Researcher 1 (A), Researcher 2 (B), or Researcher 3 (C). Red line represents the threshold between positive and negative partitions.


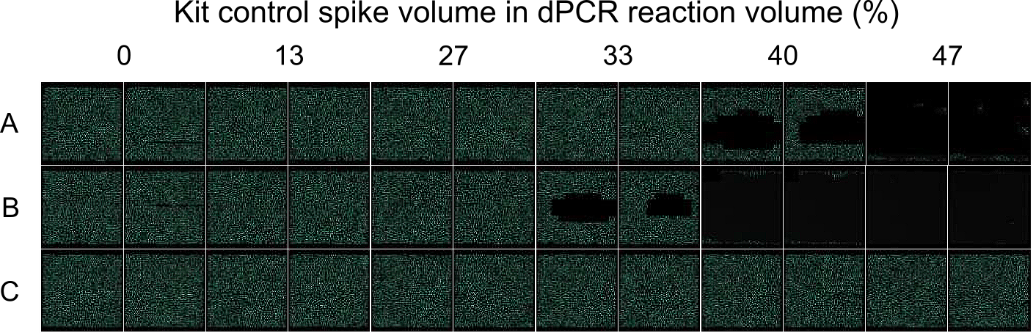


Suppl. Fig. 12. Fluorescence signal map of wells with different concentration of kit control. DNA extraction kit controls were obtained by Researcher 1 (A), Researcher 2 (B), or Researcher 3 (C).


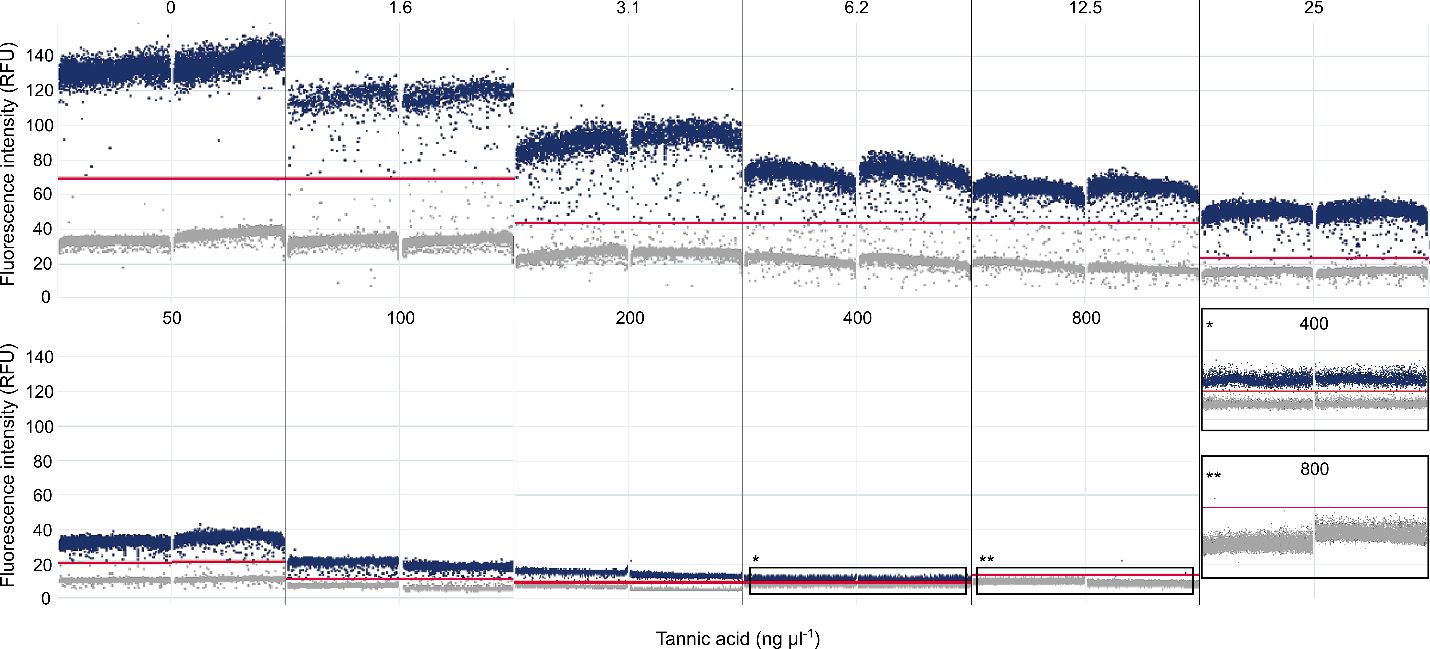


Suppl. Fig. 13. Effect of tannic acid on distribution of positive (blue dots) partitions. Red line represents a threshold between positive and negative partitions. Right bottom panels are the expanded areas indicated in black squares under * and ** showing separation of partitions at two highest concentrations tested.


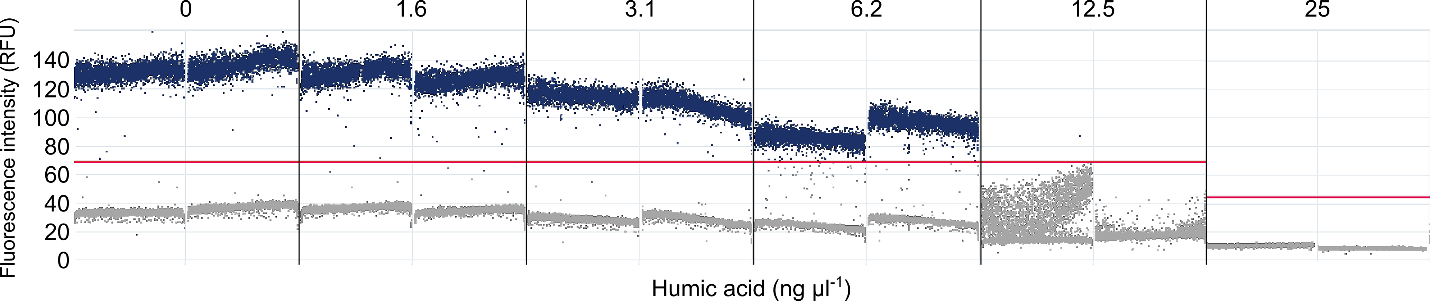


Suppl. Fig. 14. Effect of humic acid on distribution of positive (blue dots) partitions. Red line represents the threshold between positive and negative partitions.





Suppl. Fig 15. Digital PCR and qPCR performance for 341F-518R and 515F-Y-806R primer pairs on microbial community standard (mock) and environmental samples (A). The theoretical expected copy number concentration (theoretical value) is reported for mock community, synthetic standard, and groundwater samples based on the conversion of Qubit fluorometric DNA concentrations. Effect of cycle number on the ratio of intermediate to positive partitions for 341F-518R (B) and 515F-Y-806R (C) primer pairs is shown. Error bars represent standard deviation of three replicates.


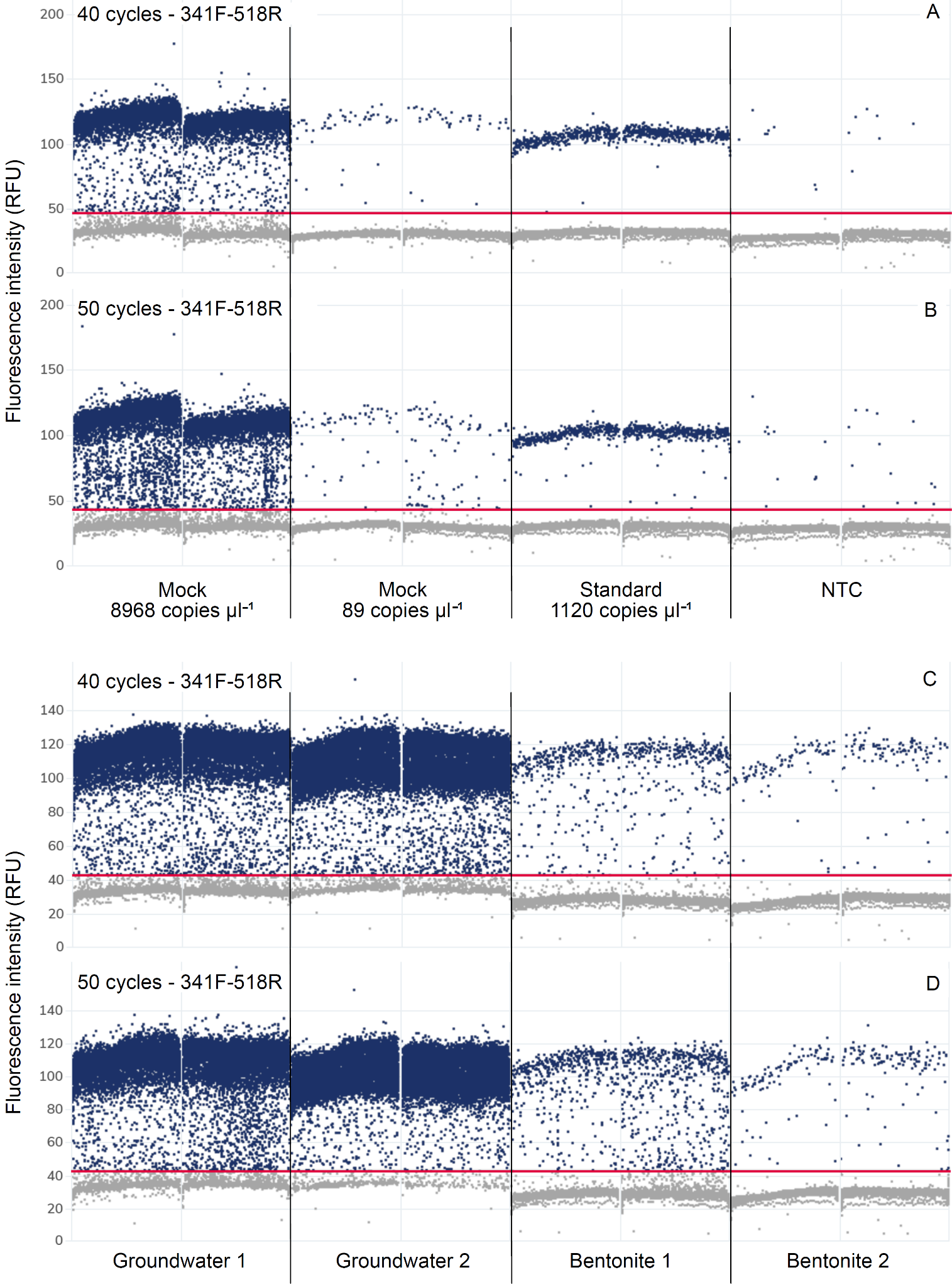


Suppl. Fig 16. Distribution of positive (blue dots) and negative (grey dots) partitions for different environmental samples and microbial community standard amplified with 341F-518R primer pair for 40 PCR (A, C) and 50 (B, D) cycles with 400 nM of primer. Red line represents the threshold between positive and negative partitions.


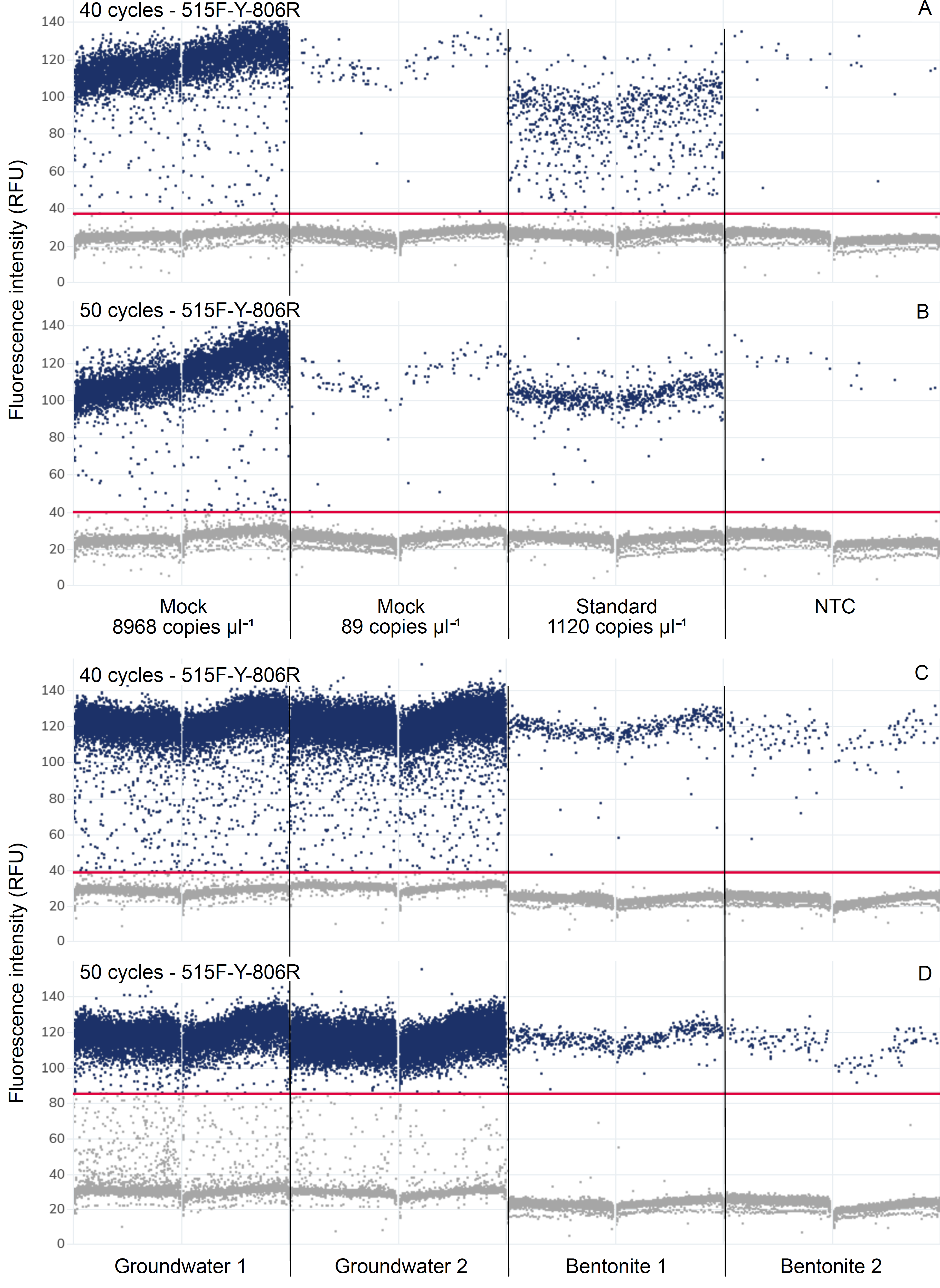


Suppl. Fig 17. Distribution of positive (blue dots) and negative (grey dots) partitions for different environmental samples and microbial community standard amplified with 515F-Y-806R primer pair for 40 PCR (A, C) and 50 (B, D) cycles with 400 nM of primer. Red line represents the threshold between positive and negative partitions.
